# The ratting of North America: A 350-year retrospective on *Rattus* species compositions and competition

**DOI:** 10.1126/sciadv.adm6755

**Published:** 2024-04-03

**Authors:** Eric Guiry, Ryan Kennedy, David Orton, Philip Armitage, John Bratten, Charles Dagneau, Shannon Dawdy, Susan deFrance, Barry Gaulton, David Givens, Olivia Hall, Anne Laberge, Michael Lavin, Henry Miller, Mary F. Minkoff, Tatiana Niculescu, Stéphane Noël, Barnet Pavao-Zuckerman, Leah Stricker, Matt Teeter, Martin Welker, Jennifer Wilkoski, Paul Szpak, Michael Buckley

**Affiliations:** ^1^ Department of Anthropology, Trent University, 1600 West Bank Dr., Peterborough, ON K9L 0G2, Canada.; ^2^ School of Archaeology and Ancient History, University of Leicester, Mayor’s Walk, Leicester LE1 7RH, UK.; ^3^ Department of Anthropology, Indiana University Bloomington, 701 E. Kirkwood Ave., Bloomington, IN 47405, USA.; ^4^ BioArCh, Department of Archaeology, University of York, Heslington, York YO10 5DD, UK.; ^5^ Independent researcher, 7 Park Court, Heath Road, Brixham TQ5 9AX, UK.; ^6^ Department of Anthropology, University of West Florida, 11000 University Pkwy, Pensacola, FL 32514, USA.; ^7^ Underwater Archaeology Team, Parks Canada, 1800 Walkley, Ottawa, ON K1H8K3, Canada.; ^8^ Department of Anthropology, University of Chicago, 1126 E 59th St, Chicago, IL 60637, USA.; ^9^ Department of Anthropology, University of Florida, Gainesville, FL 32611, USA.; ^10^ Department of Archaeology, Memorial University, Queen's College, 210 Prince Philip Dr., St. John's, NL A1B 3R6, Canada.; ^11^ Jamestown Rediscovery/Preservation Virginia, 1365 Colonial Parkway, Jamestown, VA 23081, USA.; ^12^ Département des sciences historiques, Université Laval, 1030 avenue des Sciences-Humaines, Québec, QC G1V 0A6, Canada.; ^13^ Historic St. Mary's City, St. Mary's City, MD 20686, USA.; ^14^ Florida Public Archaeology Network, 207 E Main St., Pensacola, FL 32502, USA.; ^15^ Office of Historic Alexandria/Alexandria Archaeology, 105 North Union Street, #327, Alexandria, VA 22314, USA.; ^16^ Ville de Quebec, Bureau de projet du tramway de Québec, 226-825 boul. Lebourgneuf, Québec, QC G2J 0B9, Canada.; ^17^ Department of Anthropology, University of Maryland, 4302 Chapel Lane, College Park, MD 20742, USA.; ^18^ Arizona State Museum, University of Arizona, 1013 E University Blvd. Tucson, AZ 85721, USA.; ^19^ School of Anthropology, University of Arizona, 1009 E South Campus Dr., Tucson, AZ 85721, USA.; ^20^ Colonial Williamsburg Foundation, 401 W Duke of Gloucester St, Williamsburg, VA 23185, USA.; ^21^ Manchester Institute of Biotechnology, School of Natural Sciences, The University of Manchester, 131 Princess Street, Manchester M1 7DN, UK.

## Abstract

While the impacts of black (*Rattus rattus*) and brown (*Rattus norvegicus*) rats on human society are well documented—including the spread of disease, broad-scale environmental destruction, and billions spent annually on animal control—little is known about their ecology and behavior in urban areas due to the challenges of studying animals in city environments. We use isotopic and ZooMS analysis of archaeological (1550s–1900 CE) rat remains from eastern North America to provide a large-scale framework for species arrival, interspecific competition, and dietary ecology. Brown rats arrived earlier than expected and rapidly outcompeted black rats in coastal urban areas. This replacement happened despite evidence that the two species occupy different trophic positions. Findings include the earliest molecularly confirmed brown rat in the Americas and show a deep ecological structure to how rats exploit human-structured areas, with implications for understanding urban zoonosis, rat management, and ecosystem planning as well as broader themes of rat dispersal, phylogeny, evolutionary ecology, and climate impacts.

## Teaser

Archaeological rats show dramatic temporal species shifts and diet differences, with implications for health and rat management.

## INTRODUCTION

What rats do has had, and continues to have, major consequences for humans across the globe. A number of *Rattus* species have become specially adapted to living in or near human settlements, where they exploit rich rodent habitat diversity and food opportunities (*1*, *2*). Two species, the black (*R. rattus*) and brown (*R. norvegicus*) rat, have benefited the most from their human association and have, at one time or another, become globally distributed (*3*–*5*). In the context of the Americas, black rats were first to arrive, stowing away with early European visitors and colonists, including Columbus’ initial arrival in the Caribbean in 1492 (*3*), and subsequently proliferating in settlements across the Caribbean islands and continents thereafter. Conventional wisdom, based on historical accounts, suggests that brown rats arrived later, likely by the time of American Independence in 1776 [e.g., (*3*, *6**–**11*); most cite a benchmark arrival year of 1775, following Harlan (*11*), although earlier dates have been suggested; for reviews, see (*4*, *6*)]. Aside from these dates on their arrivals, we know little else about how these commensal animals interacted, spread, and exploited the increasing availability of complex urban human-structured landscapes in the Americas (*12*, *13*). On the basis of their distribution in urban centers in North America today, rats that disembarked from these early ships soon gained a foothold in new and rich habitats across the continent. This process of rapid rat population growth and expansion had far-reaching health and ecological consequences for both people and the other species with which they shared the continent. However, documenting how rat expansion unfolded has thus far been limited to localized historical accounts and geographically disparate records of rat remains from archaeological contexts.

We know from more recent ecological research that the relationships between black and brown rats are complex and influenced by a wide range of biotic and abiotic factors (*13*–*15*). For instance, in some areas of the globe, the larger and more aggressive brown rat is thought to outcompete its smaller cousin [leading to geographical species distribution patterns driven by competitive exclusion of black rats; (*16*, *17*)], yet there are clear examples from across a variety of climates (including temperate areas) of sympatric populations with black rat dominance (*18*, *19*). This means that the ways in which these key rat species interact are strongly dependent on how their environment is structured. Moreover, while there are some general differences in the average behavioral profiles of both taxa (e.g., brown rats have a stronger affinity for in- or near-ground dwelling; black rats being more arboreal), the two species have similar, highly adaptable behavioral ranges and capabilities; thus, nonsympatric populations of either species could use the same ecological niches (*5*, *13*, *15*, *19*). In that context, and given the widespread and often intimate relationships between these taxa and humans today, developing more detailed evidence-based frameworks for the long-term spread and interspecific behaviors of black and brown rats could reveal clues for understanding fundamental dimensions of synanthropic animal ecology (*20*). Further, greater understanding of black and brown rat ecology within human systems has implications both for preventing the spread of zoonotic diseases (*21*) and for improving outcomes for the multi-billion-dollar “pest” exclusion industry (*22*).

Despite this broader importance, there have been no spatiotemporally large-scale analyses examining rodent species compositions [although see (*3*, *23*)] or diet (a prime behavioral and habitat driver) in human settlements (*12*). Taking a longer-term and geographically extensive view on these processes has potential to provide valuable insights into how these key taxa behave in response to changing anthropogenic and natural pressures over hundreds of years. This also has important implications for our understanding of how rats might respond to future changes as urban areas continue to grow and evolve, particularly in a context of broader climatic and environmental change (*24*). Although generating multi-decadal and multi-city perspectives using methods presently available to the urban ecology research community could be prohibitive in terms of both time and cost (*12*, *25*), the broad scope of archaeology, encompassing the full geography and timeframe of human-rat relationships, has potential to open new temporal windows onto rat expansion and adaptation (*3*, *4*, *20*, *26*, *27*). Archaeological assemblages in the Americas dating to the past ~530 years contain abundant invasive rodent remains. Across these various assemblages, there are, collectively, tens of thousands (and likely orders of magnitude more if future excavations are considered) *Rattus* bones, each representing a biomolecular archive with potential to shed light on the biogeography and ecology of rats at a particular point in this episode of their global spread.

**Fig. 1.**
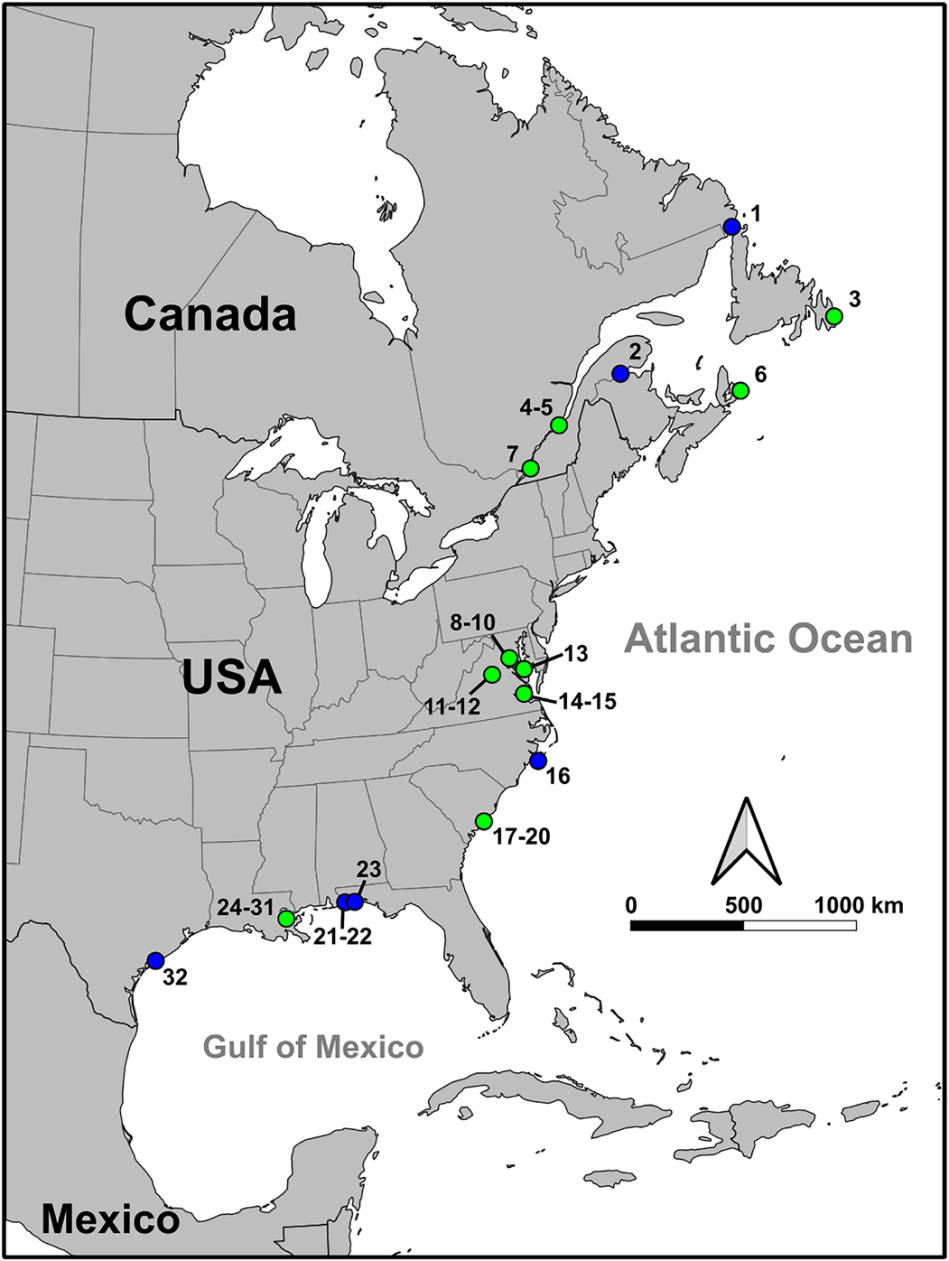
Map showing site locations. Terrestrial sites and shipwreck sites are shown by green and blue symbols, respectively. Site names (see table S1) ordered by latitude are as follows: 1. *San Jaun*; 2. *Le Machault*; 3. Ferryland; 4. rue St.-Vallier; 5. Assemblée nationale du Québec; 6. Fortress of Louisbourg; 7. St. Anne's Market; 8. Hotel Indigo; 9. House for Families; 10. South Grove; 11. Montpelier (Madison Basement); 12. Mount Pleasant; 13. St. John’s House; 14. Richneck Quarter; 15. Jamestown; 16. *Queen Anne’s Revenge*; 17. 70 Nassau St.; 18. Atlantic Wharf; 19. Heyward Washington; 20. South Adgers Wharf; 21 and 22. Emanuel Point Wrecks I and II; 23. *Rosario*; 24. Passebon Cottage; 25. 1231 Bourbon St.; 26. Ursuline Convent; 27. 936 St. Peter St.; 28. 810 Royal St.; 29. St. Anthony’s Garden; 30. 626 Bourbon St; 31. Rising Sun; 32. *La Belle*.

Here we perform a large-scale stable isotope and collagen peptide mass fingerprinting (ZooMS) analysis of the species compositions and diets of archaeological *Rattus* populations (Fig. 1) in early British and French coastal population centers around North America’s Eastern Seaboard, Canada’s Maritimes and St. Lawrence regions, and the Gulf of Mexico, spanning from the early settlement of Jamestown in 1607 through to the early 1900s. Our analyses also include rats from shipwreck contexts dating from 1550s to 1760 and geographically distributed in coastal waters from present-day Texas (USA) to Labrador (Canada). In that spatiotemporal context, our goal is to assess four questions. (a) When did brown rats first appear? (b) How did the introduction of brown rats affect the distribution of black rats? (c) Did black and brown rats compete for the same foods? (d) And lastly, were there any fundamental differences between the diets of black and brown rats regardless of whether they were sympatric? We consider the implications of our findings for the biogeography of *Rattus*’s spread around the western Atlantic World and beyond, the role of human settlements in structuring black and brown rat relationships, and recent synthesis of the differing ecologies between the two species. Our findings highlight how the diachronic study of these archaeological samples can provide ecological information that cannot be easily gathered from historical records or contemporary fieldwork.

## RESULTS

Our dataset includes rat bones from 32 archaeological sites (Fig. 1 and table S1), spanning more than 23° of latitude and from tundra (*San Juan* at Red Bay, Labrador, Canada at 51.7°N) to subtropical (*La Belle* in Matagorda Bay, Texas, USA at 51.7°N) environments. All sites are associated with coastal settlements or other concentrated areas of human activity and geographically slot into the following regional groups: New Orleans (Louisiana, USA; eight sites), Charleston (South Carolina, USA; four sites), the Chesapeake Bay (Virginia and Maryland, USA; eight sites), Quebec (Canada; three sites), and the Canadian Maritimes and Newfoundland (two sites), as well as widely distributed shipwrecks (seven sites). ZooMS and isotopic results are reported in table S2.

**Fig. 2.**
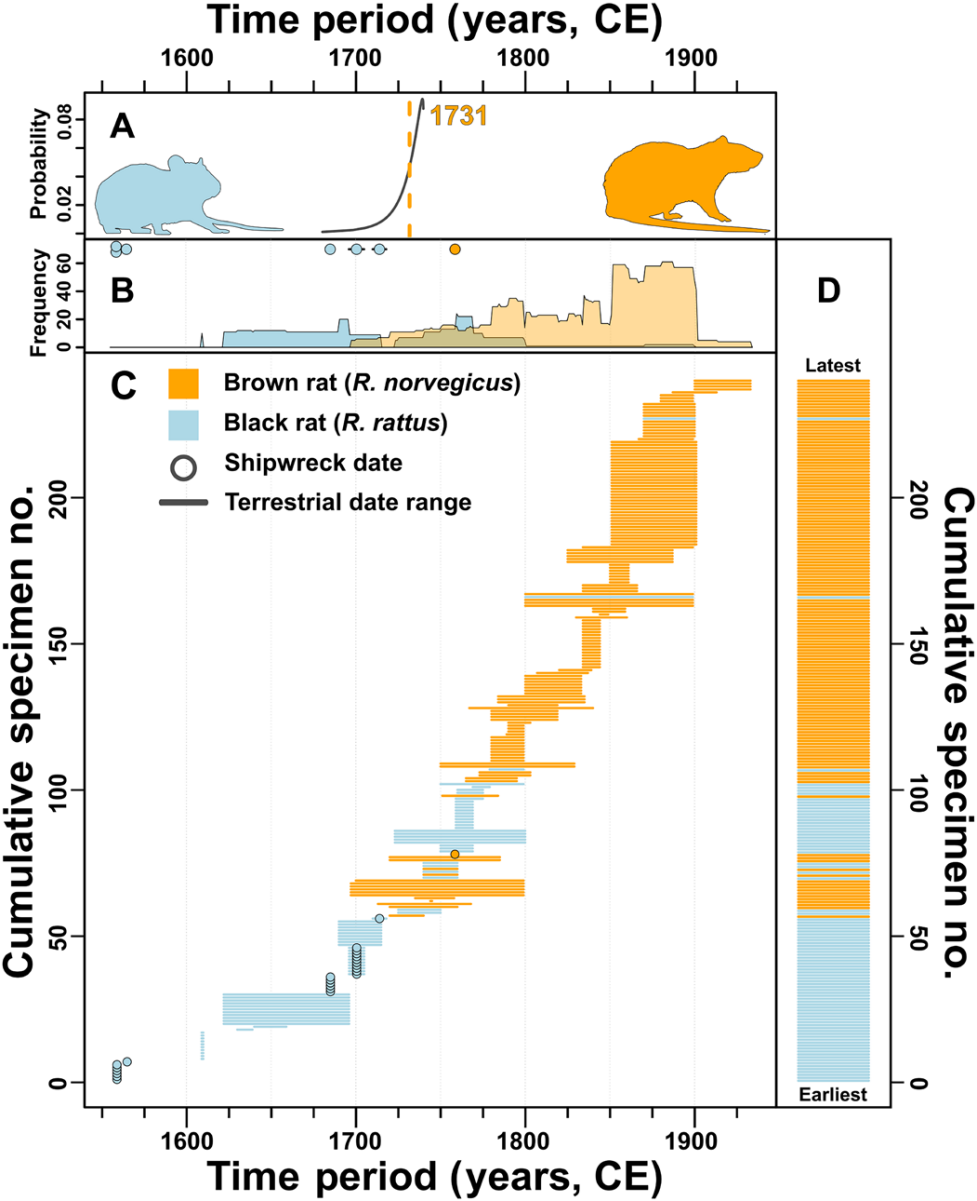
Occurrence of ZooMS-identified black and brown rats through time. (**A**) Tentative estimated dates for introduction of brown rats to North America based on OLE from our ZooMS-confirmed archaeological occurrences alone. Median estimate show by dashed line. (**B**) Frequency count of specimens from terrestrial sites with date ranges spanning each year. For reference, specimens from shipwrecks are shown as points along the top. Note that, because these data are not normalized in terms of weighting for dating certainty, the more loosely dated specimens contribute more to the area under the curve than more tightly dated specimens. (**C**) Radiator plot showing timeframe and taxon for each specimen. (**D**) Barcode plot summarizing data from (C).

We performed collagen peptide mass fingerprinting (ZooMS) to provide species identifications for rat bone specimens. ZooMS was performed on 311 samples from all 32 sites, with 269 samples producing usable mass spectra. Of these, 5 were identified as *Mus* sp., 1 was a microtine rodent (likely a vole), 88 were black rats, and 172 were brown rats. While the vast majority of samples had previously been identified only to the genus level, *Rattus* sp., based on bone morphology, 46 had been given species-level identifications including *R. norvegicus*, *R. rattus*, and *Mus musculus* (table S2). Of these, six (all postcranial bones) were reassigned to a different species based on ZooMS results (table S2). While we aimed to collect up to 20 rat specimens (averaging 7.3 samples) per site, to assess the extent to which our spatially extensive but diffuse sampling strategy might overlook presence of less abundant taxa we also undertook intensive sampling (*n* = 55) of deposits spanning much of the 1800s from one site, the St. Anne’s Market in Montréal, Canada. All ZooMS-identified samples (*n* = 54) from this site were brown rats, indicating that even when higher sample numbers are collected, we can still see a single species present at some sites. Figure 2 compares ZooMS results with timeframe (full results in table S2) and shows an initial, roughly 200-year, phase (starting with our earliest date of 1559) in which black rats are the sole or primary *Rattus* species in the region. A sharp transition from black to brown rat dominance was observed in the 1700s. Brown rats appear, at the latest, by 1760, with evidence suggesting possible introductions earlier in that century (see Discussion). Even assuming that the true date for each set of early ZooMS-identified brown rat finds falls at the end of its reported date range, we can tentatively estimate an introduction date of ~1731 using optimal linear estimation (OLE) (*28*, *29*).

**Fig. 3.**
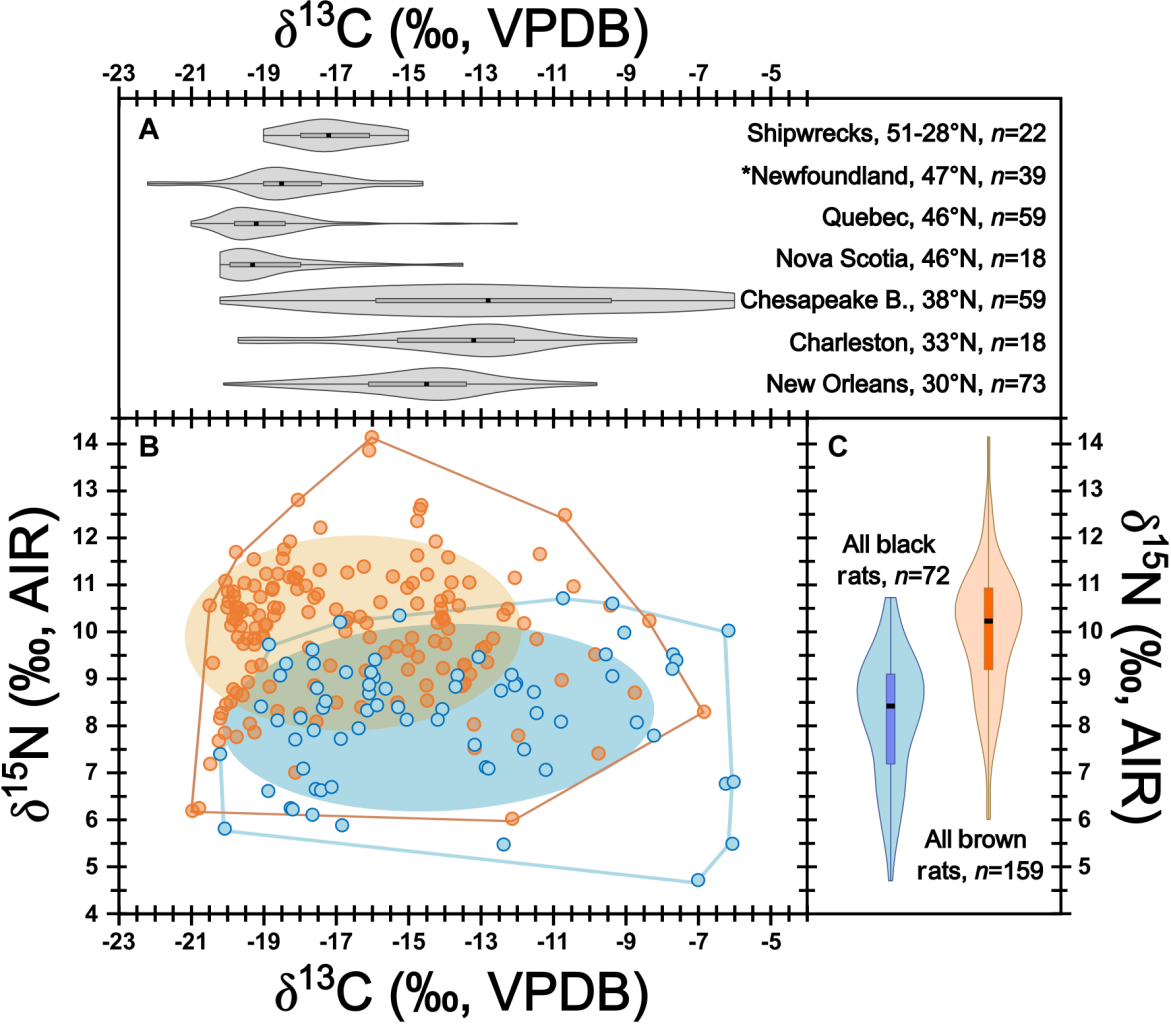
Isotopic compositions from archaeological rat bone collagen. (**A**) Violin and box plots showing kernel density for δ^13^C grouped by region and ordered by latitude (see fig. S1 for plot showing these data for δ^15^N). Occurrence of higher δ^13^C values in northern regions (Chesapeake Bay, Quebec, and Nova Scotia) reflects C_4_ influences from human agriculture (maize and/or saltmarsh use). Asterisk indicates that samples come from a fishing station where higher rat δ^13^C reflects marine food use. (**B**) Isotopic compositions, ellipses (1σ), and convex hulls for ZooMS-identified black (blue symbols) and brown (orange symbols) rats from all sites. For δ^13^C and δ^15^N, standard uncertainties for measurements were ±0.15‰ and ±0.33 ‰. (**C**) Violin and box plots showing kernel density for ZooMS-identified black and brown rat δ^15^N for all sites (see fig. S2 for plot showing these data for δ^13^C).

Isotopic analyses were performed to explore dietary trends across black and brown rat populations and included 313 samples [277 from this study plus 36 from the literature; (*15*, *30*)] from 31 sites. Of these, 299 samples passed quality control criteria, including 87 ZooMS-identified black rats and 166 ZooMS-identified brown rats (table S1). Figure 3B shows tremendous variation in isotopic compositions for both black and brown rats, as expected for generalist, highly adaptable omnivores. A breakdown of isotopic data by taxa and site is presented in table S3, with comparisons for data at all sites (*n* = 5) where both taxa were present shown in table S4.

Across all sites, δ^13^C spanned 15.5‰ (*n* = 87, mean = −14.9 ± 4.0‰; all reported SDs are 1σ unless otherwise noted) and 14.2‰ (*n* = 166, mean = −16.5 ± 3.1‰) for black and brown rats, respectively. Because this variation has more to do with regional differences in dominant plant photosynthetic pathways (see Discussion), which are linked to latitude and human agricultural choices (*31*, *32*), interspecific statistical δ^13^C comparisons should only be performed on samples grouped at the intra-regional level. The spatiotemporal distribution of sites was influenced by patterns in colonial settlement timing and urban development in coastal regions of North America. Notably, across data aggregated from all sites in each region, co-occurrences of both taxa are rare (table S3). In the Chesapeake Bay, however, where we have a larger number of early sites (and hence greater quantities of black rats), an intra-regional interspecific comparison is possible and shows no significant difference (Levene’s test, *P* = 0.344; Student’s *t* = 0.362, df = 58, *P* = 0.719) in δ^13^C between black (*n* = 45, mean = −12.5 ± 4.0‰, range = 14.2‰; Shapiro-Wilk *W* = 0.961, *P* = 0.136) and brown (*n* = 14, mean = −12.9 ± 3.2‰, range = 10.7‰; Shapiro-Wilk *W* = 0.915, *P* = 0.185) rats. The site of Ferryland in Newfoundland, Canada is also notable for having a greater abundance of both taxa. While a comparison of δ^13^C values at this site showed a significant difference (Levene’s test, *P* = 0.956; Student’s *t* = 3.870, df = 22, *P* ≤ 0.001) between black (*n* = 15, mean = −18.8 ± 1.4‰, range = 5.1‰; Shapiro-Wilk *W* = 0.898, *P* = 0.090) and brown (*n* = 8, mean = −16.5 ± 1.3‰, range = 3.4‰; Shapiro-Wilk *W* = 0.923, *P* = 0.453) rats, as a fishing station site that underwent substantial changes in sociopolitical status (affecting availability of fisheries refuse for rats) over the course of its occupation, temporal comparisons are more likely to show patterns related to economic and logistical changes at the site rather than rat behavior (*15*). For this reason, interspecific isotopic differences at Ferryland are not considered here. In that context, given the lack of interspecific difference shown among the Chesapeake Bay samples, we group δ^13^C of all rats together by region in Fig. 3A (see also table S3) to assess spatial variation in δ^13^C. A clear distinction occurs along latitudinal lines, with southern regions showing higher and more variable δ^13^C (reflecting importance of C_4_ resources) and more northerly regions showing lower and less variable δ^13^C.

As shown in Fig. 3C, in aggregate (and excluding fishing/whaling station sites of Ferryland and *San Juan* at Red Bay, as these serve to inflate differences; see Discussion and fig. S2), brown rats have a mean δ^15^N that is 1.8‰ (*n* = 159, +10.0 ± 1.4‰, range = 10.0‰; Shapiro-Wilk *W* = 0.973, *P* = 0.125) higher than black rats (*n* = 72, +8.2 ± 1.3‰, range = 8.2‰; Shapiro-Wilk *W* = 0.983, *P* = 0.053), a difference that is statistically significant (Levene’s test, *P* = 0.916; Student’s *t* = 9.286, df = 230, *P* ≤ 0.000). While co-occurrence of black and brown rats is rare, among the five sites in which we have at least one ZooMS-identified sample from each species, mean brown rat δ^15^N values are higher in all but one instance, and even in that case, the means for these two taxa differ by only 0.1‰ (table S4). Although these general differences between mean δ^15^N from black and brown rats were observed across nearly all sites, sample sizes at individual sites where there is co-occurrence are too small for statistical comparisons. As noted for δ^13^C, sufficient data from both taxa occur in the Chesapeake Bay region and, there, a comparison of δ^15^N shows a significant difference (Levene’s test, *P* = 0.013; Welch’s *t* = 10.410, df = 58, *P* ≤ 0.005), with a mean δ^15^N for brown rats (*n* = 14, +10.1 ± 1.9‰, range = 10.1‰; Shapiro-Wilk *W* = 0.886, *P* = 0.070) that is 1.8‰ higher than that for black rats (*n* = 45, +8.3 ± 1.3‰, range = 8.3‰; Shapiro-Wilk *W* = 0.967, *P* = 0.219).

## DISCUSSION

### General dietary trends

The large range in bone collagen δ^13^C for both black and brown rats reflects isotopic differences in foods available to rats over the wide latitudinal range of our study. This includes areas where food webs are based on primary production that relies on C_3_ and C_4_ photosynthetic pathways (*33*), which produce lower and higher δ^13^C values and are more prevalent in more northerly and southerly regions, respectively. In two cases, with samples coming from fishing (Ferryland, *n* = 22 ZooMS-identified samples) and whaling (*San Juan* at Red Bay, *n* = 1 ZooMS-identified sample) stations both in NL, Canada, considerable quantities of marine-derived animal protein have also influenced δ^13^C variation (*15*, *30*, *34*). Because much of the δ^13^C variation we have observed across this study is likely connected to well-documented geographical variation in the photosynthetic pathways of plant communities adapted to different climates (*31*), we do not consider variation in δ^13^C in this discussion on variation in rat behavior.

In the context of Fig. 3A, however, variation in rat δ^13^C can be clearly linked with human foodways in some cases. Particularly in northerly areas where C_4_ plants are not as naturally abundant, such as our Canadian sites and those around the Chesapeake Bay, higher rat δ^13^C is likely driven by access to human, C_4_-derived, foods such maize or naturally occurring salt hay (*Spartina* sp.), or protein from animals raised eating these foods [for review, see (*35*)]. While evidence for C_4_ feeding rats is present at very low frequencies in our Canadian sites [where maize was not a common staple; (*36*)], it is abundant at sites in the Chesapeake Bay where maize was widely grown. While beyond the scope of this paper, contextualized within robust faunal baseline datasets [under development, spanning all relevant local ecosystems, e.g., (*15*, *26*, *37*, *38*)], future site- and subregional-level interpretation of these data could provide a promising avenue for exploring ecological, social, and economic dimensions of the development of early colonial food systems (*39*–*41*). While evidence for higher δ^13^C among rats in our more southerly sites is also apparent, the natural abundance of C_4_ plants (i.e., that are not necessarily connected to particular human food strategies) in these areas makes this distinction difficult to apply without adding other isotopic data (*35*, *42*).

### The arrival and rapid rise of brown rats

Our finding that the earliest *Rattus* specimens were black rats is not surprising; however, evidence for early brown rat intrusion into these early black rat landscapes is worth closer consideration. We acknowledge that a large quantity of zooarchaeologically analyzed and unanalyzed *Rattus* remains exist in the collective historical archaeological assemblages of the Americas and that future or presently unpublished analysis may yield earlier examples. While an arrival date, based on historical accounts, of 1775 has been cited (*11*) and referred to in reference texts [e.g., (*3*, *6*–*10*)], some have suggested dates as early as the 1750s [e.g., (*43*, *44*)]. There have also been limited published identifications of brown rat bones based on skeletal morphology from archaeological deposits dating to as early as the 1740s (*3*, *23*). The brown rat’s affinity for burrowing means that even occasional early finds in terrestrial archaeological deposits that seem to be secure (i.e., nonintrusive) might be treated with caution because they could represent individuals from later time periods. This makes it difficult to establish the timing of the species’ arrival based on associated archaeological context dates of occasional finds of early brown rat bones. Moreover, the radiocarbon dating techniques that are widely applied to assess these kinds of chronological questions in archaeology and quaternary paleontology have limited capacity to resolve questions about the timing of events between the mid-1600s and 1950. Here, we have used two approaches to circumvent this issue.

The first was to investigate the species composition of rat remains recovered from shipwrecks of known ages (*45*). Since these sites typically have outstanding dating, resolved to specific years (i.e., when a ship was built and when it sank) with known travel histories (i.e., specific ports where specific amounts of time were spent) and will not include remains of intrusive (burrowing) rats, they can provide an ironclad terminus ante quem (the date before which an event must have occurred) for brown rat introduction events. Our analyses include data from seven coastal wreck sites with sinking events dating to 1559 (x2), 1565, 1686, 1705, 1718, and 1760. All, save a sample from the latest wreck, were of black rats. We identified a brown rat from the wreck of *Le Machault*, a privateer vessel launched in Bayonne (France) in 1758, that landed in Bayonne, West Africa, Bordeaux (France), and Quebec City before it sank in present-day New Brunswick at the Battle of the Restigouche on 8 July 1760 (*46*). This specimen provides the earliest biomolecularly confirmed evidence for a brown rat in the Americas and, moreover, for the species’ entry into the world’s shipping networks.

The second approach is to aggregate evidence for pre-1775 ZooMS-confirmed brown rats from terrestrial archaeological sites. The widespread appearance of brown rats in well-dated terrestrial archaeological deposits would add weight to isolated early observations of brown rats based on zooarchaeological analyses. Our data show several interesting cases. Brown rats appear in pre-1775 archaeological contexts in New Orleans (*n* = 1, 1720–1740), South Carolina (*n* = 2, 1740–1760; *n* = 4, 1750s or earlier), Virginia (*n* = 2, 1735–1758), and Nova Scotia (*n* = 1, 1744–1745; *n* = 1, 1713–1768; *n* = 1, 1751–1784; table S2). An additional sample from Newfoundland with an earlier context date range (1622–1696) is from a less secure context that may have mixed with later materials and is not considered further here. Therefore, while the vast majority of specimens that pre-date 1775 are black rats, there is a pattern demonstrating that some of these brown rat specimens could predate the definitive 1758–1760 occurrence on *Le Machault*. While we consider data from shipwrecks to be the most chronologically secure, taken at face value these onshore brown rat finds would indicate introduction before 1740, with even a conservative OLE model producing an estimate of ~1731—although this must be treated with caution given the potential dating reliability issues for terrestrial sites noted above.

If that is true, then we are left to explain how or why these early introductions articulate with what appears to be a dramatic shift in rat species representation in the 1700s (Fig. 2). By the mid-1700s, we see a significant decline in occurrence of black rats, matched by a sharp rise in the proportion of brown rats. While the archaeological dating for this transition is imprecise due to variation in chronological control for relevant contexts (spanning a wide range of different time frames from 1 to 100 years, and falling at different points in time), in aggregate, this transition appears to have occurred very quickly. Only two specimens (of 108, or 1.9%, post-1800 samples) from our entire ZooMS-identified sample show black rats occurring after 1800. More surprisingly, there are only five black rat specimens with a terminus post quem (the date after which an event must have occurred) after 1760. A conservative estimate then, taking even the earliest potential evidence we have found for brown rat introduction (1720–1740), indicates that the apparent shift from the black rats’ complete dominance to near disappearance in our dataset occurred over a span of only a few decades. This provides supporting evidence for early commentators, noting a general loss of black rats in American coastal urban centers by the 1830s (*11**,* *17**,* *47**,* *48*), by offering more detailed insight into both the timing and tempo of black and brown rat interactions and population trends. The startling pace of this transition between black and brown rat dominance merits further consideration, with wider implications for our understanding of both these species’ ecology and their impacts on human and nonhuman communities. To unpack these patterns more fully, we need to explore the isotopic evidence for black and brown rat dietary behaviors.

### Changes in prevailing rat trophic ecology

Given the wide-ranging dietary adaptability of both black and brown rats (*5*, *15*), we had expected to find a similar, large degree of overlapping variation in isotopic compositions for both species across the dataset and were therefore surprised to see variation in δ^15^N compositions that is structured around taxon (Fig. 3, B and C). While exploring these patterns, it is important to bear in mind that although rats at all sites would likely have had access to discarded marine animal protein, we make a distinction between diets of rats inhabiting sites where refuse would systematically include large amounts of marine protein, as this will have isotopic compositions driven by different processes, with dramatically different isotopic baselines. When we consider dietary variation among these species, two patterns stand out as most striking. First, when examining rat diets at nonfishing station sites, we see that brown rats on average have higher δ^15^N than black rats, indicating that brown rats occupy a higher trophic level by consuming more animal protein (*49*). We are aware that spatial and temporal differences in δ^15^N can result in variation in consumer isotopic compositions (*50*) and, for this reason, when interpreting consumer δ^15^N as evidence for trophic position, it is important to have faunal baseline δ^15^N data from the same sites and time periods to anchor interpretations [for reviews, see (*51*, *52*)]. However, in this case, we feel confident in interpreting trophic differences in the absence of such δ^15^N baseline data because the patterns occur clearly between aggregated data from each taxon across numerous sites spanning a large range of geographical and temporal contexts. To interpret these data otherwise would require an argument in which, somehow, all the sites with more brown rats just happen to also systematically have higher baseline δ^15^N values despite their wide spatial and temporal distribution.

A second pattern, supporting the first, emerges from our data when we consider isotopic variation at sites where black and brown rats co-occur and may have been sympatric. Given the rapid disappearance of black rats, sites where both are present are comparatively rare (*n* = 5). However, in nearly all cases where both species are present at the same site, brown rats have higher average δ^15^N values. Given that generalist omnivores living at the same site would likely be subject to that same baseline δ^15^N values, this provides further support for the interpretation of higher brown rat δ^15^N values as evidence for a higher trophic position.

What is perhaps most surprising is not that the two taxa differ on average in their trophic position, but that this appears to occur regardless of whether they are living sympatrically or not. One pattern in the isotopic literature on contemporary rat communities is that sympatric populations tend to have interspecific differences in their isotopic niches [e.g., (*53*–*56*)], reflecting the influence of competition between members of each species. Here, there appear to be more fundamental drivers at play, suggesting that, while there is overlap, each species tends toward a different suite of food resources even when not competing with the other. The fact that our data show that black rats on average did not routinely access the highest trophic level foods that brown rats later accessed is perplexing. It seems unlikely that higher trophic level food waste (i.e., terrestrial and aquatic animal protein) underwent a rapid, permanent, and pan-regional increase in availability in the mid-1700s, so it is likely that this difference reflects wider black rat food preferences, at least for individuals that historically lived among these settlements. In that context, the differing δ^15^N values of brown and black rats appear to represent a fundamental difference in the preferred niche of these taxa in urbanizing spaces. While quantitative urban dietary comparisons (isotopic or otherwise) for sympatric black and brown rats continue to be rare, a preference for use of more vegetation-based, less urban food resource areas has recently been observed along an urban-to-bushland spectrum for black rats in an Australian urban center [(*57*); also see (*58*)], suggesting that our spatiotemporally broader scale archaeological observations do have contemporary and potentially wider-spread analogs today.

### Further study is needed

While our findings provide insights into human-rat relationships, and expand the temporal depth of our understanding of black and brown rat distributions and diet ecology, there are several areas where further work is needed to make these findings more widely applicable for research on the biogeography, ecology, and management of rats. Analyses of additional samples from the key periods of interaction (spanning the late 1600s and 1700s) would help to better establish both the timing of brown rat introductions and the species’ rise to dominance as well as the interspecific dietary dimensions of this transition. More diversity in regional representation exploring this transition in urban centers of different sizes and with differing economic, environmental, sociopolitical, and climatic parameters would help to establish what variables are most relevant for facilitating brown rat dominance. Development of a more robust framework for the timing and tempo of the brown rats’ spread across a diverse range of urban centers would allow us to link these patterns with broader historical trends in human societies (i.e., social, economic, and technological developments) to understand the factors that lit the powder keg of brown rat expansion.

### Broader trends and implications

We have considered behavioral (e.g., relative fossorial tendencies of each species) and taphonomic (e.g., preservation and differential disposal or recovery of rat carcasses) factors that could provide alternative explanations for the dearth of black rats in later archaeological deposits, but these do not offer plausible explanations for the broad-scale imbalance. In that context, we believe that the transition between taxa represents a real change in species distribution, at least for the areas most intensively sampled here (i.e., those with denser human populations near North America’s eastern coastlines), in which black rats became and then remained comparatively scarce following the surge in brown rat populations. At the same time, it is worth bearing in mind that both historical sources (*6*, *11*, *47*, *48*, *59*) and contemporary ecological research (*60*) indicate spatiotemporal heterogeneity in the disappearance and reappearance of black rat populations in North America. While published research of the contemporary distribution of black and brown rats in North America is limited, there are clear examples of sympatric populations [e.g., (*60*, *61*)]. Our data do not necessarily suggest that black rats disappeared entirely—a number of our samples confirm that they maintained a foothold in at least some areas (e.g., Virginia and Louisiana)—but rather that they became less common to the point where they were rarely incorporated into the archaeological records we have examined.

In that context, important questions arising from our data include, “where did all the black rats go”? “What factors drove this apparent brown rat horizon”? While the isotopic compositions of black and brown rat diets do show meaningful average differences, there is still considerable overlap (Fig. 3B), likely reflecting a situation where brown rats had come to occupy part of the niche space previously used by black rats. At the same time, the isotopic evidence also shows that some of the resources that were more consistently used by black rats, particularly the lower trophic level foods, do not appear to have been as routinely used by brown rats. This suggests that black rats disappeared, or at least declined below levels routinely detectable through our approach, even though part of their isotopic niche remained unused or underused by brown rats. This apparent disconnect may, at first, seem difficult to explain, but likely involves a complex set of processes.

One potential hypothesis suggested by our data, and that may be worthy of future investigation, is related to the role of diet in the reproductive capacity for black rats at the population level. If the portion of the black rat’s niche taken by brown rats played a more important role (compared with the niche fragment left untouched by brown rats) in supporting those black rats with the greatest fecundity, this could have a cascading effect resulting in an overall population decline [e.g., (*62*, *63*)]. In the context of our isotopic data, this scenario would indicate that it was the loss of the higher trophic level end of the black rats’ diet that tipped the balance. Validation of this hypothesis could add considerable nuance to our understanding of urban black rat diets as it would suggest that, while black rats on average seek lower trophic level foods, at a population level, they could still be highly sensitive to loss of the higher trophic level portion of their preferred niche. Alternative hypotheses that cannot be ruled out include that brown rat presence served to prevent black rats from accessing other critical, non–food-related aspects of these urban habitats. For instance, such variables could include interspecific competition for territory, nest space and resources, aggressive behavioral competitive exclusion, and even predation on black rats by brown rats (*13*, *64*–*66*). Diachronic changes and spatial variation in the nature of human structuring of these variables could also be at play, including potentially sweeping infrastructural changes (the proportions of impervious surfaces, green open urban versus built-up spaces, dense urban cores versus suburban sprawls) that could more strongly influence foraging behavior and outcomes of one species relative to the other. To fully investigate the hypothesis that brown rats could have caused declining black rat reproductive outcomes due to a reduction in black rat diet quality, larger-scale and more fine-grained (capturing more data from across introduction events and from different kinds of human settlements) isotopic or other dietary analyses of sympatric archaeological or contemporary black and brown rat populations are needed.

These analyses represent a zoomed-out, spatially and temporally extensive perspective on the interactions between black and brown rats that, at the local level (settlement or subsettlement), likely involved highly variable context-dependent drivers, contingent on the availability of particular kinds of structures, resources, and pathways for population connectivity. While further investigation is therefore needed to fully contextualize these broader-scale trends (as outlined above, see the previous section), this initial insight into the spatiotemporally broad-scale dynamics of *Rattus* distributions and dietary behavior in North America also has a wide range of potentially useful implications for contemporary ecology and strategies for mitigating the impacts of rats on human and nonhuman communities.

1) Zoonosis. To the extent that prevailing differences in the ecology of black versus brown rats can create different circumstances and opportunities for the transmission and spread of zoonotic diseases to and from humans, our data suggest that those associated with the brown rat likely would have had more frequent occurrence in urban centers in this region of the world over the last 250 years.

2) “Pest” exclusion. To the extent that food availability can be controlled by policy for guiding human food waste disposal patterns in urban spaces, those focused on curbing brown rat access to animal protein sources should have the largest impact on constraining this species’ preferred niche.

3) *Rattus* evolution and behavior. A wide range of interspecific competitive pressures can drive species behavior, creating selective pressures that contribute to evolutionary and behavioral variation across space and time (*14*, *20*, *27*). The rapid loss of black rats, and continued existence of part of their niche (no longer used by either *Rattus* species), suggests that black rats exert less selective pressure on brown rats than one might expect (given their ability to use similar resource bases). This suggests that selective pressures on brown rats are likely not heavily driven by black rat competition in these urban spaces.

4) Climate impacts. Our data capture black-to-brown-rat transition events at sites spanning more than 17° of latitude, as far north as Ferryland (Newfoundland, Canada) and south as New Orleans (Louisiana, USA). This suggests that the ecological drivers of the patterns we have observed are insulated to some extent from climatic variation and, in turn, that competitive pressure on black and brown rats can act independently from climate in urban spaces in coastal regions of this area of the world.

5) Population origins and demographics research. Our data suggest that brown rat populations in many coastal urban areas of North America likely represent longer established or more stable commensal rodent populations. The decline in black rats identified from our data raises questions around the origins and longevity of any black rat populations found across this region following the introduction of brown rats. For instance, our results suggest that extirpation events may have been common and that contemporary black rat populations in these areas could be the result of recolonization(s). Genetic work on contemporary black rat populations is needed to explore rates of population turnover, periods of population decline, and multiple recolonization events. Work exploring genetic patterns in contemporary black rats should anticipate that they are examining populations that, in some cases, may not be continuous from the region’s founding black rat populations and may not have been a dominant rat population in an area for up to 250 years.

6) Brown rat dispersal. These data add important reference points to our framework for understanding the pathways and timing of the brown rat’s spread beyond the Americas, including Europe. Despite its comparative recency, the global dispersal of brown rats is poorly understood in terms of routes, timings, and trigger factors (*25*), with numerous contradictory and often un-evidenced dates and narratives in the literature. While phylogenetics suggests that Western Europe was a stepping stone in dispersal to Atlantic North America (*67*), a convergence of dates between early historical documentation of brown rats in Europe (*68*, *69*) and our biomolecular evidence from North America indicates that the offset between arrival in these two continents was decades at most—emphasizing rapid radiation by maritime routes. The entry of brown rats into European colonial shipping networks—which by the later 1700s linked all inhabited continents—may have been a key event. In the context of the Americas, beginning in 1565, direct Spanish maritime trade from Manilla (Philippines) to Mexico (crossing land from Acapulco to Veracruz), which connected with ports in the Caribbean en route to Europe, also opened an earlier, direct Asian conduit for the introduction of rats to Atlantic North America. Genetic analysis of securely dated archaeological and historical specimens is necessary to disentangle a rapid yet likely complex dispersal process.

7) Competition with indigenous animals. The niche left unused or underused when black rats declined could have represented an opportunity for other taxa to take up residence in or near human settlements. The introduction of brown rats could result in competitive release, opening up space for other commensal and nondomesticate, synanthropic animals that had previously been in direct competition with black rats. In that context, the brown rat’s introduction could have had a positive impact on urban fauna species richness, at least for taxa that seek lower trophic level foods. In turn, and from another perspective, given the rapid rise of brown rats, it is worth considering the possibility that black rats had formed part of a “primary succession” of colonial invasive species that cleared the way of would-be competitors, creating the conditions needed for brown rats to take up residence more quickly in urban settings. Isotopic analyses of rodents at pre-contact Indigenous settlements suggest that native micro-mammals already filled niches offered by human urban spaces before the introduction of *Rattus* (*70*). In that context, further analyses of noninvasive faunas that could have competed for the same resources could contextualize this and other questions about where and how this succession of invasive rat species fits within broader human ecosystems.

It is also worth reflecting on how these results highlight the importance of incorporating archaeological perspectives into understandings of animal behavior in urban spaces (*71*). Archaeology is uniquely positioned not only to contextualize broader scale trends in the origins, development, and trajectories of synanthropic animal behaviors that are driven by the evolution of urban environments [e.g., structures, food opportunities, and social attitudes; e.g., (*3*, *4*, *20*, *26*, *27*)] but also to provide access to a vast biomolecular archive with potential to rewrite the history of a variety of human-animal relationships [e.g., *72*, *73*–*75*)]. In the context of rat ecology, while there has been an explosion of publications in the recent literature, relatively little attention is given to the relative impacts of the two species (i.e., the implications of whether black, brown, or both rat species are present) on human health, wildlife, and the economy. Among the many contemporary ecological syntheses published in the past 5 years [e.g., (*25*, *76*–*79*)], for instance, none review evidence in relation to taxonomic occurrence across broader spatial (e.g., urban versus rural, populations density, regional climate, and governance geography) or temporal scales [though see (*27*)] or how this parameter affects human health, wildlife outcomes, or the economic costs of sharing urban spaces with rodents. In this context, most discussion has focused either on rats irrespective of taxon or on the dominant brown rat. There is, therefore, a risk of viewing rats as fungible actors when, in fact, as our results highlight, there is deep ecological structure to which species is able to more effectively exploit human settlements. This has implications for ongoing management at all scales (e.g., spatial and socioeconomic). Although this disconnect, in part, reflects inherent challenges in doing research on rats in urban spaces (*12*, *25*), it seems as much driven by siloing of knowledge that partitions the past and present/future along disciplinary lines, separating archaeological and contemporary ecological research communities. Those that have incorporated a longer view (*20*, *26*, *27*) explicitly call for research investigating the conditions under which both taxa coexist and the factors that offer competitive advantages to one or the other or that serve as tipping points for range expansions.

In that context, and viewed more broadly, if the wider goal of urban ecology is to plan for a future where urban environments are habitats for people and animals to thrive together (*24*, *80*–*83*), then basing those plans on perspectives from smaller scale, recent timeframes could put us at a disadvantage. Moreover, given the challenges of excluding rodents from urban spaces (*78*), rats will almost certainly have a place in that planned-for-future. It therefore stands to reason that building urban ecological plans on retrospectives that span hundreds or thousands of years, incorporating the tremendous range of human urban development, rather than observations from the past few decades, will create more robust strategies and approaches for urban planning. This means that archaeological perspectives have an essential role to play in future strategies for living with rats in urban spaces. Furthermore, on the heels of a global pandemic, and the renewed appreciation this has generated for the value of ecologically situated understandings of the origins and spread of zoonotic diseases, contributions of such archaeological perspectives could have benefits that go well beyond urban planning.

## MATERIALS AND METHODS

### Experimental design

Bones remodel slowly in comparison with the tissues typically analyzed in isotopic studies of contemporary animal populations [e.g., hair, epidermis, blood, and muscle; (*84*, *85*)]. For this reason, the isotopic composition of bone collagen will reflect a longer-term average of the foods an animal has consumed over the course of its life. In the context of isotopic research on archaeological rat bones, comparatively short lifespans mean that bone collagen isotope compositions will offer temporal perspectives that integrate diet over a period of between several months and a couple of years [weighted more toward periods of rapid growth; (*15*)].

Here we have aimed to collect samples from sites spanning a wide range of human population densities, from cities to smaller settlements. Recent work by Guiry and Buckley (*26*) shows that brown rat diets can vary in relation to human population density particularly when comparing isotopic variation between urban and rural areas. However, in the context of this study, even the sites with the least urban development (e.g., rural plantations, fishing stations, fortresses, and ships) represented much higher human population densities than the threshold for “rural” (i.e., small, isolated farmsteads with one or two buildings) used in Guiry and Buckley’s (*26*) study. For this reason, we do not expect variation in our data to be a function of settlement density.

### Sample selection

Samples were selected from 25 terrestrial and 7 underwater (shipwreck) sites with the goal of creating a dataset covering a broad spatiotemporal area. While some sites are not located directly on the marine coastline, in the broader geographical context of eastern North America, all sites are considered to have been close (within 10 or 20 km) to the coast and/or ports that regularly received maritime commerce. Given the large numbers of sites included here, sampling occurred in stages over several years. While the initial stages of sampling efforts sought to include samples from all time periods, the latter stages aimed to zoom in temporally on rats dating to the 1700s. We took this approach because results from initial sampling indicated that the black-to-brown rat transition occurred around then and, further, this would increase chances of observing situations in which these species were sympatric.

In most cases, samples come from specimens that were zooarchaeologically identified only to the genus level (i.e., as *Rattus* sp.); however, a small number of samples are from specimens where faunal analysts had offered species identifications (i.e., *R. rattus* and *R. norvegicus*). In these cases, we made no attempt to preferentially select one species over the other. Where possible, sample selection proceeded with a goal of sampling the largest number of distinct individuals per context by targeting repeating elements (i.e., if a context had more right tibiae than other kinds of *Rattus* sp. bones, these would be selected). This approach minimizes potential for sampling the same individual multiple times. In general, we sought to sample adult individuals, although in a few cases younger individuals (based on size and epiphyseal fusion) were sampled.

### Isotopic analyses

Samples were demineralized in 0.5 M HCl at room temperature (solutions refreshed daily until reactions were complete) and then rinsed to neutrality in type 1 water (resistivity = 18 megohm·cm). Samples were then treated with 0.1 M NaOH in an ultrasonic bath (solutions refreshed every 20 min until visual evidence for reactions ceased) to remove base soluble contaminants (primarily humic acids) and then rinsed to neutrality again in type 1 water. Samples were then refluxed in a 10^−3^ HCl (pH 3) solution in an oven at 65°C for 36 hours. Refluxed samples were then centrifuged, and the solubilized fraction was pipetted to a fresh tube, frozen, and lyophilized.

Subsamples of collagen (0.5 mg) were analyzed using an elemental analyser coupled to an isotope ratio mass spectrometer (EA-IRMS). Analyses included replication of 29% of samples. Isotopic compositions were calibrated using two- or three-point calibration curves anchored to international standards. Accuracy was monitored with analyses of a suite of check standards spanning the range of expected sample isotopic compositions. Accepted (calibration) or long-term average (check) isotopic compositions and SDs for all standard reference materials are available in table S5. Averages and SDs for calibration standards (table S6), check standards (table S7), and sample replicates (table S8) are also reported in the Supplementary Materials. For δ^13^C and δ^15^N, systematic errors [μ_(bias)_] were ±0.10‰ and ±0.22‰, respectively; random errors [μR_(w)_] were ±0.12‰ and ±0.25‰, respectively; and standard uncertainties were ±0.15‰ and ±0.33‰, respectively. The integrity of isotopic data was evaluated using well-established collagen quality control criteria including carbon (>13.8%) and nitrogen (>4.0%) elemental concentrations (*86*) and liberal C:N criteria (*87*).

Statistical comparisons of isotopic compositions were performed using PAST version 4.13. For each group, normality of distribution was assessed using a Shapiro-Wilk test. For groups that were not normally distributed, a Mann-Whitney *U* test was used to compare means. For groups that were normally distributed, a Levene’s test was used to assess homogeneity of variance. A Welch’s *t* test (if variances unequal) or a Student’s *t* test (if variances equal) was then used to compare means.

### ZooMS

Bone collagen samples (~1 to 2 mg) were resuspended with 100 μl of 50 mM ammonium bicarbonate and digested with 0.4 μg of sequencing-grade trypsin (Promega, UK) overnight at 37°C. Following 1:10 dilution in 0.1% trifluoroacetic acid, digested collagen was then cocrystallized with an equal volume of α-cyano hydroxycinnamic acid (10 mg/ml) (Sigma-Aldrich, UK) and left to dry for subsequent peptide mass fingerprint analysis using a Bruker Rapiflex Matrix Assisted Laser Desorption Ionization Time of Flight (MALDI-ToF) mass spectrometer collecting up to 20,000 laser acquisitions over the mass/charge ratio (*m*/*z*) range 700 to 3700. Resultant spectra were then compared to those of *R. rattus* and *R. norvegicus* (*88*).

### Optimal linear estimation

Likely introduction dates of brown rats to North America were estimated via OLE (*29*) using the sExtinct package in R v.4.3.1 (*89*, *90*). See (*28*) for discussion of this technique’s applicability to introductions and to archaeological dating. To produce a conservative estimate, we assumed the true date for each set of confirmed brown rat finds to be the final year within its reported date range.

## Supplementary Materials

### This PDF file includes:

Tables S1 to S8 Figs. S1 and S2 References
