# Supplementary material for: The ratting of North America: A 350-year retrospective on Rattus species compositions and competition
Source: Sci Adv. 2024 Apr 3;10(14):eadm6755. doi: 10.1126/sciadv.adm6755 (PMC10990262; doi:10.1126/sciadv.adm6755)
Supplement: Supplementary file 1 — Tables S1 to S8 Figs. S1 and S2 References [file sciadv.adm6755_sm.pdf]

Science Advances
AAAS

Supplementary Materials for

The ratting of North America: A 350-year retrospective on *Rattus* species compositions and competition

Eric Guiry *et al.*

Corresponding author: Eric Guiry, eric.guiry@trentu.ca; Michael Buckley, m.buckley@manchester.ac.uk

Sci. Adv. **10**, eadm6755 (2024)
DOI: 10.1126/sciadv.adm6755

This PDF file includes:

Tables S1 to S8
Figs. S1 and S2
References

Table S1. Details for sites included in this study. Asterisk indicates fishing or whaling sites where rat diets will be more strongly influenced by marine protein.

Site Name	Site Number	City/Area	State/Province	Type	Collective Sample Date Range	Samples (*n*=)
1231 Bourbon St.	NA	New Orleans	Louisiana, USA	Terrestrial	1780s to 1819	4
626 Bourbon St.	16OR736	New Orleans	Louisiana, USA	Terrestrial	1790s to 1890s	9
810 Royal St.	16OR706	New Orleans	Louisiana, USA	Terrestrial	1720 to late 1800s	14
Passebon Cottage	16OR142	New Orleans	Louisiana, USA	Terrestrial	1830s to 1900	8
Rising Sun	16OR225	New Orleans	Louisiana, USA	Terrestrial	1720s-1913	9
St. Anthony's Garden	16OR443	New Orleans	Louisiana, USA	Terrestrial	Early 1800s	3
Ursuline Convent	16OR49	New Orleans	Louisiana, USA	Terrestrial	1720 to late 1800s	9
936 St. Peter St.	16OR737	New Orleans	Louisiana, USA	Terrestrial	1850s to 1900	20
70 Nassau St.	38CH1600	Charleston	South Carolina, USA	Terrestrial	c. 1900	5
Atlantic Wharf	38CH1606	Charleston	South Carolina, USA	Terrestrial	1750s to 1820s	5
Heyward Washington	38CH108	Charleston	South Carolina, USA	Terrestrial	1740 to 1760	5
South Adgers Wharf	38CH 2291	Charleston	South Carolina, USA	Terrestrial	1750s or earlier	5
St. John’s House	18ST1-23	Chesapeake	Maryland, USA	Terrestrial	1690 to 1715	9
Hotel Indigo	44AX229	Chesapeake	Virginia, USA	Terrestrial	1780 to early 1800s	12
House for Families (Mount Vernon)	44FX762/40+47	Chesapeake	Virginia, USA	Terrestrial	1759 to 1790s	13
Jamestown	NA	Chesapeake	Virginia, USA	Terrestrial	1609 to 1610	10
Mount Pleasant (Montpelier)	44OR219	Chesapeake	Virginia, USA	Terrestrial	1723 to 1800	6
Madison Basement (Montpelier)	44OR249	Chesapeake	Virginia, USA	Terrestrial	1800s	4
Williamsburg, Richneck Quarter	44WB52	Chesapeake	Virginia, USA	Terrestrial	1725 to 1775	9
South Grove (Mount Vernon)	44FX762/17	Chesapeake	Virginia, USA	Terrestrial	1735 to 1800s	4
Assemblée nationale du Québec	CeEt-740	Quebec City	Quebec, Canada	Terrestrial	1740 to c. 1780	3
rue St.-Vallier	CeEt-745	Quebec City	Quebec, Canada	Terrestrial	1784 to 1835	3
St. Anne Market	BjFj-4	Quebec City	Quebec, Canada	Terrestrial	1834 to 1901	55
Ferryland	CgAf-2	Maritimes/Newfoundland	Newfoundland, Canada	Terrestrial*	1622 to 1800	36
Fortress of Louisbourg	NA	Maritimes/Newfoundland	Nova Scotia, Canada	Terrestrial	1713 to 1784	18
San Jaun	24M8N10	Maritimes/Newfoundland	Newfoundland, Canada	Underwater*	Sank 1565	1

Le Machault	2M	Maritimes/Newfoundland	New Brunswick, Canada	Underwater	Built 1757, sank 1760	2
Queen Anne's Revenge	31CR314	North Carolina	North Carolina, USA	Underwater	Built 1710, sank 1718	2
Emanuel Point Wreck 1	8ES1980	Gulf of Mexico	Florida, USA	Underwater	Sank 1559	3
Emanuel Point Wreck 2	8ES3345	Gulf of Mexico	Florida, USA	Underwater	Sank 1559	5
Rosario	8ES1905	Gulf of Mexico	Florida, USA	Underwater	Built 1696, sank 1705	10
La Belle	41MG86	Gulf of Mexico	Texas, USA	Underwater	Built 1684, sank 1686	10

Table S2. Isotopic, elemental concentration, and ZooMS results as well as context details for all samples. Region Groups: 1=New Orleans, 2= Charleston, 3= Chesapeake Bay, 4= Nova Scotia, 5= Quebec, 6= Newfoundland, 7= widely distributed shipwrecks. Liberal quality control tests for assessing integrity of isotopic data (Lib. QC) follow (*87*): 1= pass, 2= fail. Stable isotope data from Ferryland samples with MARC and SUBC prefixes are from (*30*) and (*15*), respectively. Stable isotope analyses were not performed on specimens from the *Queen Anne's Revenge*. ZooMS analyses were not performed on the four Ferryland samples with the IUBC prefix.

Sample Number	Catalog Number	Site Name	Region Group	Time Frame	Bone	Side	Zooarch. ID	ZooMS ID	$\delta^{13}C$ (‰)	$\delta^{15}N$ (‰)	%C	%N	C:N	Lib. QC	Context Information
HEAL-1360	T3	1231 Bourbon St.	1	1780s-1819	Mandible	Right	*Rattus* sp.	*R. norvegicus*	-18.0	8.2	39.5	14.9	3.08	1	Well, Context 4, Level JK Buckets, Lot 7, Spec. 0.003
HEAL-1368	T11	1231 Bourbon St.	1	1780s-1819	Femur	Right	*Rattus* sp.	*R. norvegicus*	-16.2	9.1	41.4	15.6	3.10	1	Unit N1W1, Context 14, Level N, 15-30 cm below surface, Lot 13, Spec. 0.004
HEAL-1370	T13	1231 Bourbon St.	1	1780s-1819	Mandible	Right	*Rattus* sp.	*R. norvegicus*	-18.6	8.3	40.7	15.4	3.07	1	East room, Context ST1, Level 12-35 cm below surface, Lot 18, Spec. 0.002
HEAL-1384	T27	1231 Bourbon St.	1	1780s-1819	Tibia	Right	*Rattus* sp.	*R. norvegicus*	-11.8	10.1	39.9	15.3	3.04	1	Unit N1E7.5, Level STRI/II, 0-30 cm below surface, Lot STRI/II, Spec. 0.002
TEAL-6973	21.003	626 Bourbon St.	1	1840s-1850s	Innominate	Left	*Rattus* sp.	*R. norvegicus*	-14.1	10.6	41.7	15.1	3.21	1	Unit N2E17, Context 13, Level 1
TEAL-6974	31.002	626 Bourbon St.	1	1790s-1810s	Femur	Left	*Rattus* sp.	*R. norvegicus*	-18.1	7.0	41.3	14.9	3.23	1	Unit N1E18, Context 17, Level 1
TEAL-6976	34.004	626 Bourbon St.	1	1880s-1890s	Mandible	Right	*Rattus* sp.	*R. norvegicus*	-13.9	10.0	40.7	14.7	3.23	1	Unit N1E17, Context 4, Level 1
TEAL-6977	34.005	626 Bourbon St.	1	1880s-1890s	Mandible	Right	*Rattus* sp.	*R. norvegicus*	-12.4	10.3	41.2	14.9	3.23	1	Unit N1E17, Context 4, Level 1
TEAL-6978	34.006	626 Bourbon St.	1	1880s-1890s	Mandible	Right	*Rattus* sp.	*R. norvegicus*	-14.9	9.8	41.0	14.7	3.25	1	Unit N1E17, Context 4, Level 1
TEAL-6979	35.002	626 Bourbon St.	1	1840s-1850s	Premaxilla	Right	*Rattus* sp.	*R. norvegicus*	-14.1	10.3	41.7	15.0	3.25	1	Unit N1E17, Context 6/13, Level 2
TEAL-6980	41.012	626 Bourbon St.	1	1820s-1830s	Mandible	Right	*Rattus* sp.	*R. norvegicus*	-11.5	9.8	41.1	14.9	3.21	1	Unit N1E17, Context 16, Level 3
TEAL-6981	42.006	626 Bourbon St.	1	Early 1800s	Tibia	Left	*Rattus* sp.	*R. norvegicus*	-14.1	10.3	41.3	15.0	3.21	1	Unit N1E17, Context 16, Level 4
TEAL-6975	34.001	626 Bourbon St.	1	1880s-1890s	Innominate	Right	*Rattus* sp.	Unsuccessful	-13.1	10.0	41.4	15.0	3.22	1	Unit N1E17, Context 4, Level 1
TEAL-6943	11.085	810 Royal St.	1	1790s-1803	Humerus	Right	*Rattus* sp.	*R. norvegicus*	-15.3	9.7	40.9	14.8	3.23	1	Unit N1W0, Context 3, Level 1
TEAL-6945	32.003	810 Royal St.	1	1790s	Tibia	Left	*Rattus* sp.	*R. norvegicus*	-16.1	13.8	41.1	14.8	3.23	1	Unit N3W17, Context 22, Level 1
TEAL-6946	44.002	810 Royal St.	1	1789-1790s	Tibia	Right	*Rattus* sp.	*R. norvegicus*	-10.4	10.9	41.6	14.8	3.27	1	Unit N3W17, Context 27, Level 1
TEAL-6947	49.001	810 Royal St.	1	Late 1800s	Femur	Left	*Rattus* sp.	*R. norvegicus*	-12.1	11.1	41.1	14.7	3.25	1	Unit N1W2, Context 1, Level 1
TEAL-6950	75.015	810 Royal St.	1	1790s	Tibia	Left	*Rattus* sp.	*R. norvegicus*	-17.6	8.8	40.5	14.5	3.24	1	Unit N6W1-3, Context 44, Level 2
TEAL-6951	79.035	810 Royal St.	1	1790s	Tibia	Left	*Rattus* sp.	*R. norvegicus*	-13.4	9.3	40.8	14.8	3.22	1	Unit N6W1-3, Context 44, Level 3
TEAL-6952	157.001	810 Royal St.	1	Mid 1800s	Tibia	Right	*Rattus* sp.	*R. norvegicus*	-13.9	10.7	41.1	14.8	3.24	1	Unit N7W1-2, Context 91, Level 2
TEAL-6953	157.002	810 Royal St.	1	Mid 1800s	Tibia	Right	*Rattus* sp.	*R. norvegicus*	-17.2	9.2	41.2	14.8	3.25	1	Unit N7W1-2, Context 91, Level 2
TEAL-6957	256.013	810 Royal St.	1	c. 1788	Tibia	Left	*Rattus* sp.	*R. norvegicus*	-15.0	9.2	41.2	14.9	3.22	1	Unit N1W16, Context 148, Level 1
TEAL-6958	259.025	810 Royal St.	1	c. 1788	Tibia	Right	*Rattus* sp.	*R. norvegicus*	-14.5	8.8	40.2	14.5	3.23	1	Unit N1W16, Context 148, Level 2

TEAL-6959	260.001	810 Royal St.	1	1720-1740	Mandible	Right	*Rattus* sp.	*R. norvegicus*	-16.1	10.2	40.0	14.5	3.23	1	Unit N4W1, Context 146, Level 3
TEAL-6948	49.002	810 Royal St.	1	Late 1800s	Femur	Left	*Rattus* sp.	Unsuccessful	-14.0	10.3	41.6	14.9	3.26	1	Unit N1W2, Context 1, Level 1
TEAL-6954	158.001	810 Royal St.	1	1840s-1860s	Femur	Right	*Rattus* sp.	Unsuccessful	-15.6	11.2	41.4	14.9	3.23	1	Unit N7W1-2, Context 85, Level 1
TEAL-6955	161.003	810 Royal St.	1	1789-1790s	Tibia	Right	*Rattus* sp.	Unsuccessful	-14.8	5.4	40.1	14.3	3.28	1	Unit N4W15, Context 27, Level 2
TEAL-7015	3.002	936 St. Peter St.	1	1870-1900	Femur	Left	*Rattus* sp.	*R. norvegicus*	-12.9	9.6	41.5	14.9	3.25	1	Unit N2E0, Context 1/2
TEAL-7016	3.003	936 St. Peter St.	1	1870-1900	Femur	Left	*Rattus* sp.	*R. norvegicus*	-15.0	9.6	41.6	15.0	3.24	1	Unit N2E0, Context 1/2
TEAL-7021	7.004	936 St. Peter St.	1	1870-1900	Mandible	Left	*Rattus* sp.	*R. norvegicus*	-14.9	11.0	41.2	14.6	3.30	1	Unit N2E0, Context 1/2
TEAL-7022	7.005	936 St. Peter St.	1	1870-1900	Mandible	Left	*Rattus* sp.	*R. norvegicus*	-14.7	11.6	41.5	15.0	3.22	1	Unit N2E0, Context 1/2
TEAL-7023	7.006	936 St. Peter St.	1	1870-1900	Mandible	Left	*Rattus* sp.	*R. norvegicus*	-14.0	9.7	41.3	14.6	3.30	1	Unit N2E0, Context 1/2
TEAL-7024	7.007	936 St. Peter St.	1	1870-1900	Mandible	Left	*Rattus* sp.	*R. norvegicus*	-14.1	10.5	40.5	14.4	3.28	1	Unit N2E0, Context 1/2
TEAL-7025	17.001	936 St. Peter St.	1	1850s-1861	Mandible	Left	*Rattus* sp.	*R. norvegicus*	-13.3	11.0	40.8	14.6	3.25	1	Unit N2E0, Context 9, Level 3
TEAL-7026	17.002	936 St. Peter St.	1	1850s-1861	Mandible	Left	*Rattus* sp.	*R. norvegicus*	-15.4	11.1	37.7	13.4	3.27	1	Unit N2E0, Context 9, Level 3
TEAL-7027	18.001	936 St. Peter St.	1	1850s-1861	Tibia	Left	*Rattus* sp.	*R. norvegicus*	-14.0	10.2	41.0	14.7	3.26	1	Unit N2E0, Context 10, Level 1
TEAL-7029	18.003	936 St. Peter St.	1	1850s-1861	Tibia	Left	*Rattus* sp.	*R. norvegicus*	-14.5	11.2	40.9	14.8	3.23	1	Unit N2E2, Context 10, Level 1
TEAL-7030	21.003	936 St. Peter St.	1	1870-1900	Mandible	Left	*Rattus* sp.	*R. norvegicus*	-12.8	9.3	41.2	14.7	3.26	1	Unit N2E3, Context 2, Level 2
TEAL-7031	31.307	936 St. Peter St.	1	1850s-1861	Tibia	Left	*Rattus* sp.	*R. norvegicus*	-14.7	9.4	40.0	14.3	3.27	1	Unit N2E4, Context 9, Level 3
TEAL-7032	31.308A	936 St. Peter St.	1	1850s-1861	Tibia	Left	*Rattus* sp.	*R. norvegicus*	-15.0	10.9	41.5	14.7	3.30	1	Unit N2E5, Context 9, Level 3
TEAL-7033	31.308B	936 St. Peter St.	1	1850s-1861	Tibia	Left	*Rattus* sp.	*R. norvegicus*	-13.6	10.5	40.3	14.2	3.30	1	Unit N2E6, Context 9, Level 3
TEAL-7119	3.006	936 St. Peter St.	1	1870-1900	Femur	Left	*Rattus* sp.	*R. rattus*	-17.6	9.3	42.3	15.3	3.22	1	Unit N2E0, Context 1/2
TEAL-7017	3.004	936 St. Peter St.	1	1870-1900	Femur	Left	*Rattus* sp.	Unsuccessful	-14.3	8.1	41.6	14.8	3.27	1	Unit N2E0, Context 1/2
TEAL-7018	3.005	936 St. Peter St.	1	1870-1900	Femur	Left	*Rattus* sp.	Unsuccessful	-13.9	9.9	41.4	13.8	3.49	2	Unit N2E0, Context 1/2
TEAL-7019	5.001	936 St. Peter St.	1	1870-1900	Mandible	Left	*Rattus* sp.	Unsuccessful	-15.2	9.9	41.2	14.4	3.35	1	Unit NA, Context 1/2
TEAL-7020	5.002	936 St. Peter St.	1	1870-1900	Mandible	Left	*Rattus* sp.	Unsuccessful	-15.6	10.7	41.2	14.7	3.27	1	Unit NA, Context 1/2
TEAL-7028	18.002	936 St. Peter St.	1	1850s-1861	Tibia	Left	*Rattus* sp.	Unsuccessful	-15.2	10.4	41.5	14.7	3.29	1	Unit N2E1, Context 10, Level 1
TEAL-7037	16.003	Passebon Cottage	1	1870-1900	Femur	Right	*Rattus* sp.	*R. norvegicus*	-14.2	10.2	22.8	8.2	3.25	1	Unit N2E10, Context 3, Level 2
TEAL-7038	16.004	Passebon Cottage	1	1870-1900	Femur	Right	*Rattus* sp.	*R. norvegicus*	-10.7	12.4	41.4	15.1	3.21	1	Unit N2E11, Context 3, Level 2
TEAL-7043	44.005	Passebon Cottage	1	1870-1900	Femur	Left	*Rattus* sp.	*R. norvegicus*	-13.3	9.1	41.5	15.0	3.24	1	Unit N2W4, Context 5, Level 1
TEAL-7044	50.003	Passebon Cottage	1	1870-1900	Femur	Right	*Rattus* sp.	*R. norvegicus*	-13.4	8.9	41.0	14.8	3.23	1	Unit N1W3, Context 5, Level 2
TEAL-7039	16.005	Passebon Cottage	1	1870-1900	Femur	Right	*Rattus* sp.	Unsuccessful	-13.9	10.3	40.6	14.8	3.19	1	Unit N2E12, Context 3, Level 2
TEAL-7040	17.002	Passebon Cottage	1	1870-1900	Femur	Right	*Rattus* sp.	Unsuccessful	-13.3	9.0	40.8	14.7	3.22	1	Unit NA, Context 5

TEAL-7041	28.001	Passebon Cottage	1	1830s-1840s	Femur	Right	*Rattus* sp.	Unsuccessful	-12.5	10.0	40.7	14.6	3.25	1	Unit N2W2, Context 11, Level 1
TEAL-7042	44.004	Passebon Cottage	1	1870-1900	Femur	Right	*Rattus* sp.	Unsuccessful	-11.6	10.4	41.0	15.0	3.19	1	Unit N2W4, Context 5, Level 1
TEAL-2859	None	Rising Sun	1	1825-1887	Tibia	Left	*Rattus* sp.	*R. norvegicus*	-17.7	9.6	35.3	13.5	3.05	1	Lot 3, Exc. U. 1, Strata C, Feature 1, Lev. 2 (SW 1/4), larger individual
TEAL-2860	None	Rising Sun	1	1825-1887	Tibia	Left	*Rattus* sp.	*R. norvegicus*	-14.0	9.2	30.8	10.7	3.36	1	Lot 3, Exc. U. 1, Strata C, Feature 1, Lev. 2 (SW 1/4), smaller individual
TEAL-2861	None	Rising Sun	1	1825-1887	Tibia	Left	*Rattus* sp.	*R. norvegicus*	-15.8	9.6	39.5	12.6	3.66	2	Lot 4, Exc. U. 1, Strata NA, Feature 1, Lev. 1 (45-50)
TEAL-2862	None	Rising Sun	1	1825-1887	Tibia	Left	*Rattus* sp.	*R. norvegicus*	-16.2	11.4	38.6	13.7	3.29	1	Lot 6, Exc. U. 1, Strata D, Feature 2, Lev. 1 (55-65), smaller individual
TEAL-2863	None	Rising Sun	1	1825-1887	Tibia	Right	*Rattus* sp.	*R. norvegicus*	-19.0	9.8	39.1	13.7	3.31	1	Lot 6, Exc. U. 1, Strata D, Feature 2, Lev. 1 (55-65), larger individual
TEAL-2864	None	Rising Sun	1	ca. 1822	Tibia	Left	*Rattus* sp.	*R. norvegicus*	-13.0	9.6	40.7	14.0	3.38	1	Lot 11, Exc. U. 7, Strata 6, Feature 3, Lev. 1
TEAL-2865	None	Rising Sun	1	1887-1913	Tibia	Left	*Rattus* sp.	*R. norvegicus*	-17.0	10.4	39.1	13.5	3.38	1	Lot 111, Exc. U. 9, Strata A, Lev. 1 (26-36)
TEAL-2866	None	Rising Sun	1	1720s-1785	Tibia	Left	*Rattus* sp.	*R. norvegicus*	-16.7	11.2	40.1	14.4	3.25	1	Lot 115, Exc. U. 9, Strata B, Lev. 1 (43), smaller individual
TEAL-2867	None	Rising Sun	1	1720s-1785	Tibia	Right	*Rattus* sp.	*R. norvegicus*	-19.0	9.3	39.1	14.0	3.25	1	Lot 115, Exc. U. 9, Strata B, Lev. 1 (44-53), larger individual
TEAL-2856	2766a	St. Anthony's Garden	1	Early 1800s	Femur	Right	*Rattus* sp.	*R. norvegicus*	-17.0	8.5	34.7	11.9	3.40	1	Lot 122, Exc. U. 1x-Box 10, Context 51, Level 1, Depth below surface 100-110cm
TEAL-2857	2766b	St. Anthony's Garden	1	Early 1800s	Femur	Right	*Rattus* sp.	*R. norvegicus*	-12.8	9.9	38.8	12.7	3.56	2	Lot 122, Exc. U. 1x-Box 10, Context 51, Level 1, Depth below surface 100-110cm
TEAL-2858	2766c	St. Anthony's Garden	1	Early 1800s	Femur	Right	*Rattus* sp.	*R. norvegicus*	-9.8	9.5	38.2	13.6	3.27	1	Lot 122, Exc. U. 1x-Box 10, Context 51, Level 1, Depth below surface 100-110cm
TEAL-2868	None	Ursuline Convent	1	Early 1900s	Humerus	Right	*Rattus* sp.	*R. norvegicus*	-16.7	10.2	41.8	15.0	3.25	1	Lot 25, Exc. U. 2, Context 4, Lev. 1, larger individual
TEAL-2869	None	Ursuline Convent	1	Early 1900s	Humerus	Right	*Rattus* sp.	*R. norvegicus*	-14.3	9.8	42.4	15.3	3.23	1	Lot 25, Exc. U. 2, Context 4, Lev. 1, smaller individual
TEAL-2870	None	Ursuline Convent	1	Mid-Late 1800s	Tibia	Left	*Rattus* sp.	*R. norvegicus*	-12.2	10.5	41.7	15.0	3.24	1	Lot 27, Exc. U. 2, Context 6, Lev. 1
TEAL-2871	None	Ursuline Convent	1	Mid 1800s	Tibia	Right	*Rattus* sp.	*R. norvegicus*	-16.0	9.9	39.6	14.1	3.27	1	Lot 31, Exc. U. 2, Context 11, Lev. 1, Depth below surface 50-60cm
TEAL-2872	None	Ursuline Convent	1	Late 1700s-1840	Ulna	Right	*Rattus* sp.	*R. norvegicus*	-13.8	11.0	39.2	14.1	3.24	1	Lot 38, Exc. U. 1, Context 16, Lev. 1, Depth below surface 44-54cm
TEAL-2873	None	Ursuline Convent	1	ca. 1845	Femur	Right	*Rattus* sp.	*R. norvegicus*	-20.1	8.2	41.1	14.8	3.24	1	Lot 55, Exc. U. 1, Context 23, Lev. 1, Depth below surface 64cm
TEAL-2874	None	Ursuline Convent	1	1720-1760	Mandible	Left	*Rattus* sp.	*R. norvegicus*	-13.5	8.8	41.0	14.6	3.26	1	Lot 59, Exc. U. 2, Context 29, Lev. 1, Depth below surface 91-95cm
TEAL-2875	None	Ursuline Convent	1	Early 1900s	Tibia	Left	*Rattus* sp.	*R. norvegicus*	-15.8	10.6	42.5	15.3	3.23	1	Lot 65, Exc. U. 3, Context 4, Lev. 1, Depth below surface 19-26cm
TEAL-2876	None	Ursuline Convent	1	Early 1900s	Femur	Left	*Rattus* sp.	*R. norvegicus*	-16.7	10.0	41.8	14.9	3.27	1	Lot 24, Exc. U. 2, Context 2, Lev. 1, Depth below surface 8-17cm
TEAL-10884	1320003	70 Nassau St.	2	ca. 1900	Cranium	Axial	*R. norvegicus*	Unsuccessful	-12.6	10.0	40.6	14.5	3.27	1	Feature 1, Well
TEAL-10885	1320001-1	70 Nassau St.	2	ca. 1900	Mandible	Right	*Rattus* sp.	Unsuccessful	-13.6	10.4	43.7	15.1	3.38	1	Feature 1, Well
TEAL-10886	1320001-2	70 Nassau St.	2	ca. 1900	Mandible	Right	*Rattus* sp.	Unsuccessful	-13.3	10.5	43.2	14.9	3.37	1	Feature 1, Well
TEAL-10887	1320001-3	70 Nassau St.	2	ca. 1900	Mandible	Right	*Rattus* sp.	Unsuccessful	-12.7	9.9	43.1	15.2	3.31	1	Feature 1, Well
TEAL-10888	1320001-4	70 Nassau St.	2	ca. 1900	Mandible	Right	*Rattus* sp.	Unsuccessful	-15.2	9.4	42.8	14.4	3.48	1	Feature 1, Well
TEAL-10880	00610017	Atlantic Wharf	2	1750-1820s	Mandible	Left	*Rattus* sp.	*R. norvegicus*	-10.8	8.9	43.3	15.5	3.27	1	Feature F56
TEAL-10883	00610103	Atlantic Wharf	2	1750-1820s	Mandible	Right	*Rattus* sp.	*R. norvegicus*	-12.1	6.0	43.6	15.9	3.20	1	Feature S23, Trench TP1, Zone 10, Level 4

TEAL-10879	00610008	Atlantic Wharf	2	1750-1820s	Mandible	Right	*Rattus* sp.	Unsuccessful	-11.8	7.1	43.6	15.7	3.24	1	Feature FS7
TEAL-10881	00610023	Atlantic Wharf	2	1750-1820s	Mandible	Right	*Rattus* sp.	Unsuccessful	-14.3	9.0	38.0	12.5	3.55	2	Feature S17
TEAL-10882	00610036	Atlantic Wharf	2	1750-1820s	Mandible	Right	*Rattus* sp.	Unsuccessful							Feature S18
HEAL-92	ARL28050.43	Heyward Washington	2	1740-1760	Humerus	Right	*Rattus* sp.	*R. norvegicus*	-8.7	8.7	41.0	14.9	3.21	1	Unit 2, FS no. 53
HEAL-96	ARL28111.70	Heyward Washington	2	1740-1760	Mandible	Right	*R. norvegicus*	*R. norvegicus*	-14.5	8.5	35.5	12.8	3.24	1	Unit 5, Zone 5, FS no. 114
HEAL-93	ARL28149.41	Heyward Washington	2	1740-1760	Maxilla	Left	*Rattus* sp.	*R. rattus*	-18.6	8.1	41.3	15.0	3.22	1	Unit 6, FS no. 152
HEAL-94	ARL28110.42	Heyward Washington	2	1740-1760	Mandible	Right	*R. rattus*	*R. rattus*	-15.9	9.0	40.7	14.5	3.27	1	Units 4 and 5, FS no. 113
HEAL-95	ARL28111.71	Heyward Washington	2	1740-1760	Mandible	Left	*R. rattus*	*R. rattus*	-12.0	8.9	39.9	14.4	3.22	1	Unit 5, FS no. 114
HEAL-88	ARL48181.1	South Adgers Wharf	2	1750s or earlier	Femur	Right	*Rattus* sp.	*R. norvegicus*	-15.6	9.5	40.5	14.6	3.23	1	Unit N340E345, Zone 10, FS no. 284
HEAL-89	ARL48180.4	South Adgers Wharf	2	1750s or earlier	Femur	Left	*Rattus* sp.	*R. norvegicus*	-19.7	7.7	41.4	15.0	3.21	1	Unit N345E325, Zone 10, FS no. 283
HEAL-90	ARL48178.2	South Adgers Wharf	2	1750s or earlier	Femur	Right	*Rattus* sp.	*R. norvegicus*	-12.7	9.8	39.9	14.6	3.19	1	Unit N345E325, Zone 10, FS no. 281
HEAL-91	ARL48177.4	South Adgers Wharf	2	1750s or earlier	Mandible	Left	*Rattus* sp.	*R. norvegicus*	-13.2	7.5	40.3	14.5	3.24	1	Unit N345E325, Zone 10, FS no. 280
HEAL-87	ARL48170.4	South Adgers Wharf	2	1750s or earlier	Femur	Left	*Rattus* sp.	*R. rattus*	-13.2	7.6	39.3	14.2	3.22	1	Unit N340E325, Zone 10, FS no. 273
HEAL-539	20	Hotel Indigo	3	Early 1800s	Femur	Right	*R. norvegicus*	*R. norvegicus*	-11.2	7.9	41.5	10.9	4.44	2	Site Area 72FP, Context F35, Wbisc
HEAL-540	84	Hotel Indigo	3	Early 1800s	Humerus	Left	*R. norvegicus*	*R. norvegicus*	-9.4	10.5	39.8	13.7	3.39	1	Site Area 72FP, Context F37, Ebisc, Feature Fill L4
HEAL-541	229	Hotel Indigo	3	1780-1799	Femur	Right	*R. norvegicus*	*R. norvegicus*	-11.4	11.6	39.9	14.3	3.25	1	Site Area 72FP, Context F36, SBisc Feature Fill L1
HEAL-542	546	Hotel Indigo	3	1780-1799	Humerus	Right	*R. norvegicus*	*R. norvegicus*	-14.6	12.6	39.8	14.2	3.28	1	Site Area 72FP, Context F56, NBisc L3, Feature Fill 2
HEAL-543	547	Hotel Indigo	3	1780-1799	Humerus	Right	*R. norvegicus*	*R. norvegicus*	-16.3	8.4	39.5	14.0	3.29	1	Site Area 72FP, Context F56, NBisc L3, Feature Fill 2
HEAL-544	715	Hotel Indigo	3	1780-1799	Humerus	Right	*R. norvegicus*	*R. norvegicus*	-17.5	8.1	40.7	14.6	3.25	1	Site Area 72FP, Context F56, SBisc L7, Feature Fill 4
HEAL-545	789	Hotel Indigo	3	1780-1799	Femur	Right	*R. norvegicus*	*R. norvegicus*	-14.7	12.6	36.8	13.1	3.28	1	Site Area 72FP, Context F56, SBisc L8, Feature Fill 5
HEAL-546	863	Hotel Indigo	3	1780-1799	Femur	Left	*R. norvegicus*	*R. norvegicus*	6.0	10.5	16.6	14.0	1.37	2	Site Area 72FP, Context F56m SBisc L5, Feature Fill 3
HEAL-547	19	Hotel Indigo	3	Early 1800s	Tibia	Right	*Rattus* sp.	*R. norvegicus*	-6.8	8.3	40.8	14.6	3.26	1	Site Area 72FP, Context F35, Ebisc
HEAL-548	718	Hotel Indigo	3	1780-1799	Tibia	Left	*Rattus* sp.	*R. norvegicus*	-14.7	10.6	40.0	14.4	3.24	1	Site Area 72FP, Context F56, SBisc L7 Feature Fill 4
HEAL-549	719	Hotel Indigo	3	1780-1799	Tibia	Left	*Rattus* sp.	*R. norvegicus*	-14.7	12.3	38.0	13.7	3.24	1	Site Area 72FP, Context F56, SBisc L7, Feature Fill 4
HEAL-550	979	Hotel Indigo	3	1780-1799	Tibia	Left	*Rattus* sp.	*R. norvegicus*	-14.2	11.9	41.3	14.6	3.29	1	Site Area 72FP, Context F56, SBisc L4, Feature Fill 3
HEAL-491	4328	House for Families	3	1779-1790s	Humerus	Left	*Rattus* sp.	*Mus* sp.	-9.0	9.2	38.3	13.2	3.38	1	Context 40A-8, Site 72AD, Phase C
HEAL-480	414	House for Families	3	1759-1769	Femur	Left	*Rattus* sp.	*R. rattus*	-12.1	9.0	40.3	14.1	3.33	1	Context 040FF, Site 72AD, Phase A
HEAL-481	536	House for Families	3	1759-1769	Femur	Left	*Rattus* sp.	*R. rattus*	-16.8	5.9	40.9	14.5	3.30	1	Context 040EE, Site 72AD, Phase A
HEAL-482	782	House for Families	3	1779-1790s	Ulna	Left	*Rattus* sp.	*R. rattus*	-7.6	9.4	39.9	14.6	3.20	1	Context 0040G, Site 72AD, Phase C
HEAL-483	2336	House for Families	3	1759-1769	Innominate	Left	*R. rattus*, cf	*R. rattus*	-12.1	8.8	37.6	13.6	3.23	1	Context 0047X, Site 72AD, Phase A

HEAL-484	2324	House for Families	3	1769-1779	Mandible	Right	*R. rattus*, cf	*R. rattus*	-16.4	7.9	40.8	14.8	3.22	1	Context 0040E, Site 72AD, Phase B
HEAL-485	4125	House for Families	3	1759-1769	Humerus	Right	*Rattus* sp.	*R. rattus*	-11.5	8.7	39.7	14.4	3.22	1	Context 47X-8, Site 72AD, Phase A
HEAL-486	4181	House for Families	3	1759-1769	Humerus	Right	*Rattus* sp.	*R. rattus*	-9.4	10.6	36.1	13.0	3.23	1	Context 47AA-8, Site 72AD, Phase A
HEAL-487	4276a	House for Families	3	1759-1769	Femur	Left	*Rattus* sp.	*R. rattus*	-9.5	9.5	36.1	12.9	3.25	1	Context 40FF-8, Site 72AD, Phase A
HEAL-488	4276b	House for Families	3	1759-1769	Femur	Left	*Rattus* sp.	*R. rattus*	-7.7	9.5	39.7	14.4	3.21	1	Context 40FF-8, Site 72AD, Phase A
HEAL-489	4276c	House for Families	3	1759-1769	Femur	Left	*Rattus* sp.	*R. rattus*	-12.4	8.7	38.6	13.9	3.23	1	Context 40FF-8, Site 72AD, Phase A
HEAL-490	4276d	House for Families	3	1759-1769	Femur	Left	*Rattus* sp.	*R. rattus*	-9.0	9.9	37.3	13.7	3.18	1	Context 40FF-8, Site 72AD, Phase A
HEAL-538	4126	House for Families	3	1759-1769	Humerus	Right	*Rattus* sp.	*R. rattus*	-9.4	9.0	36.4	12.7	3.35	1	Context 47X-8, Site 72AD, Phase A
TEAL-1739	1853	Jamestown	3	1609-1610	Humerus	Left	*Rattus* sp.	*R. rattus*	-16.1	8.7	45.2	16.4	3.21	1	Context Pit 1, JR no. 2H, UB no. 1853
TEAL-1740	3852	Jamestown	3	1609-1610	Mandible	Right	*R. rattus*	*R. rattus*	-18.1	7.7	46.5	17.0	3.20	1	Context Pit 3, Seq B, JR no. 69F, UB no. 3852
TEAL-1741	10554	Jamestown	3	1609-1610	Mandible	Left	*R. rattus*	*R. rattus*	-16.1	8.3	43.6	15.8	3.22	1	Context Pit 3, Seq B, JR no. 124F, UB no. 10554
TEAL-1742	18795	Jamestown	3	1609-1610	Tibia	Right	*Rattus* sp.	*R. rattus*	-11.8	7.5	36.9	13.3	3.24	1	Context Pit 5, JR no. 731M, UB no. 18795
TEAL-1743	18876	Jamestown	3	1609-1610	Tibia	Right	*Rattus* sp.	*R. rattus*	-10.8	8.1	41.6	15.0	3.24	1	Context Pit 5, JR no. 731M, UB no. 18876
TEAL-1744	14291	Jamestown	3	1609-1610	Tibia	Right	*Rattus* sp.	*R. rattus*	-18.4	9.3	45.1	16.5	3.20	1	Context Pit 8, JR no. 1795B, UB no. 14291
TEAL-1745	16720	Jamestown	3	1609-1610	Femur	Right	*Rattus* sp.	*R. rattus*	-17.5	8.8	45.1	16.4	3.21	1	Context Jane Cellar, JR no. 3081C, UB no. 16720
TEAL-1746	16380	Jamestown	3	1609-1610	Femur	Right	*Rattus* sp.	*R. rattus*	-15.9	8.4	45.2	16.6	3.17	1	Context Jane Cellar, JR no. 3081F, UB no. 16380
TEAL-1747	16652	Jamestown	3	1609-1610	Femur	Right	*Rattus* sp.	*R. rattus*	-18.0	8.1	45.2	16.3	3.22	1	Context Jane Cellar, JR no. 3081K, UB no. 16652
TEAL-1748	16655	Jamestown	3	1609-1610	Tibia	Left	*Rattus* sp.	*R. rattus*	-17.3	8.4	46.0	16.9	3.18	1	Context Jane Cellar, JR no. 3081K, UB no. 16655
TEAL-9968	336.FM a	Madison Basement	3	1800s	Radius	Right	*Rattus* sp.	*R. norvegicus*	-15.3	8.5	45.6	16.4	3.25	1	INVENTORY no. 336.FM, MT/PROVEN no. 405.F4A
TEAL-9970	336.EC	Madison Basement	3	1800s	Femur	Left	*Rattus* sp.	*R. norvegicus*	-9.7	7.4	46.4	16.8	3.22	1	INVENTORY no. 336.EC, MT/PROVEN no. 405.F4A
TEAL-9971	336.EQ	Madison Basement	3	1800s	Femur	Right	*Rattus* sp.	*R. norvegicus*	-13.2	8.0	45.7	16.4	3.25	1	INVENTORY no. 336.EQ, MT/PROVEN no. 405.F4A
TEAL-9969	336.FM b	Madison Basement	3	1800s	Ulna	Right	*Rattus* sp.	*R. rattus*	-6.2	6.7	44.8	16.2	3.22	1	INVENTORY no. 336.FM, MT/PROVEN no. 405.F4A
TEAL-9963	1067	Mount Pleasant	3	1723-1800	Humerus	Left	*R. rattus*	*R. rattus*	-14.1	8.3	44.7	16.0	3.26	1	UA no. 00100234, INVENTORY no. 1067, MT/PROVEN no. OP1 L42 Lot24
TEAL-9964	1125a	Mount Pleasant	3	1723-1800	Innominate	Left	*R. rattus*	*R. rattus*	-12.8	7.1	45.9	16.6	3.22	1	UA no. 00100454, INVENTORY no. 1125, MT/PROVEN no. OP1 L42 Lot3
TEAL-9965	1125b	Mount Pleasant	3	1723-1800	Cranium	Left	*R. rattus*	*R. rattus*	-12.9	7.1	44.4	16.1	3.22	1	UA no. 00100454, INVENTORY no. 1125, MT/PROVEN no. OP1 L42 Lot30
TEAL-9966	1214	Mount Pleasant	3	1723-1800	Radius	Right	*Rattus* sp.	*R. rattus*	-14.2	8.1	43.7	15.8	3.22	1	UA no. 00100210, INVENTORY no. 1214, MT/PROVEN no. OP1 L42 Lot34
TEAL-9967	432	Mount Pleasant	3	1723-1800	Femur	Left	*R. rattus*	*R. rattus*	-11.5	8.2	45.5	16.3	3.25	1	UA no. 00100444, INVENTORY no. 432, MT/PROVEN no. MT201.G
TEAL-9960	973	Mount Pleasant	3	1723-1800	Tibia	Right	*R. rattus*	Unsuccessful	-8.2	7.4	43.4	14.7	3.44	2	UA no. 00100382, INVENTORY no. 973, MT/PROVEN no. OP1 L42 Lot19
HEAL-627	18277	Richneck Quarter	3	1750-1769	Mandible	Left	*Rattus* sp.	Microtine	-19.6	3.8	35.3	12.7	3.25	1	Context AL00128, Feature F07, 68AL, 1009-AL00128-FLT--00084
HEAL-624	11576	Richneck Quarter	3	1725-1750	Mandible	Left	*Rattus* sp.	*Mus* sp.	-7.6	8.0	38.6	13.8	3.27	1	Context AL00090, Feature F05a, 68AL, 1009-AL00090-FLT--00355

HEAL-623	17528	Richneck Quarter	3	1725-1750	Femur	Right	*Rattus* sp.	*R. rattus*	-11.2	7.0	41.9	15.4	3.18	1	Context AL00133, Feature F10, 68AL, 1009-AL00133-FLT--00492
HEAL-625	11081	Richneck Quarter	3	1725-1750	Mandible	Left	*Rattus* sp.	*R. rattus*	-7.7	9.2	37.5	13.5	3.23	1	Context AL00105, Feature F05a, 68AL, 1009-AL00105-FLT--00746
HEAL-626	17997	Richneck Quarter	3	1750-1769	Femur	Right	*Rattus* sp.	*R. rattus*	-20.2	7.4	38.6	13.9	3.23	1	Context AL00036, Feature F10, 68AL, 1009-AL00036-FLT--00169
HEAL-628	17098	Richneck Quarter	3	1750-1769	Mandible	Left	*Rattus* sp.	*R. rattus*	-8.7	8.1	37.7	13.6	3.23	1	Context AL00165, Feature F21, 68AL, 1009-AL00165-FLT--00201
HEAL-629	17177	Richneck Quarter	3	1750-1769	Mandible	Left	*Rattus* sp.	*R. rattus*	-13.1	9.4	35.4	12.5	3.30	1	Context AL00171, Feature F21, 68AL, 1009-AL00171-FLT--00121
HEAL-630	15995	Richneck Quarter	3	1760-1775	Mandible	Left	*Rattus* sp.	*R. rattus*	-6.2	10.0	38.1	14.0	3.18	1	Context AL00042, Feature F21, 68AL, 1009-AL00042-FLT--00292
HEAL-631	16105	Richneck Quarter	3	1760-1775	Mandible	Left	*Rattus* sp.	*R. rattus*	-13.6	9.0	39.0	14.2	3.19	1	Context AL00162, Feature F21, 68AL, 1009-AL00162-FLT--00190
HEAL-441	14816	South Grove	3	1759-1775	Radius	Right	*Rattus* sp. cf	*Mus* sp.	-15.8	8.3	39.8	14.3	3.24	1	Context 349KK, Phase 2, Site 72, Area BN
HEAL-440	16002	South Grove	3	1800s	Femur	Left	*Rattus* sp.	*R. norvegicus*	-8.3	10.2	40.0	14.4	3.24	1	Context 329T, Phase 4, Site 72, Area BN
HEAL-442	14708	South Grove	3	1735-1758	Femur	No Data	*Rattus* sp.	*R. norvegicus*							Context 348SS, Phase 1, Site 72, Area BN
HEAL-443	15448	South Grove	3	1759-1775	Humerus	Left	*Rattus* sp.	*R. rattus*	-8.2	7.8	40.4	14.6	3.24	1	Context 329JJ, Phase 2, Site 72, Area BN
HEAL-2307	2K/KI	St. John’s House	3	1690-1715	Tibia	Right	*Rattus* sp.	*R. rattus*	-12.4	5.5	41.4	14.9	3.24	1	Cellar, 2K/KI
HEAL-2308	3A/AEG	St. John’s House	3	1690-1715	Tibia	Right	*Rattus* sp.	*R. rattus*	-13.7	8.8	40.7	14.3	3.31	1	Cellar, 3A/AEG
HEAL-2309	4/FYT	St. John’s House	3	1690-1715	Tibia	Right	*Rattus* sp.	*R. rattus*	-7.0	4.7	41.5	14.9	3.26	1	Cellar, 4/FYT
HEAL-2310	4D/MB	St. John’s House	3	1690-1715	Tibia	Right	*Rattus* sp.	*R. rattus*	-6.0	6.8	41.0	14.4	3.31	1	Cellar, 4D/MB
HEAL-2311	4G/JJ	St. John’s House	3	1690-1715	Tibia	Left	*Rattus* sp.	*R. rattus*	-15.2	10.3	42.0	15.0	3.25	1	Cellar, 4G/JJ
HEAL-2312	80E/VR	St. John’s House	3	1690-1715	Tibia	Right	*Rattus* sp.	*R. rattus*	-10.7	10.7	40.9	14.4	3.31	1	Cellar, 80E/VR
HEAL-2313	80F/EK	St. John’s House	3	1690-1715	Tibia	Right	*Rattus* sp.	*R. rattus*	-18.8	9.7	41.5	14.8	3.27	1	Celler, 80F/EK
HEAL-2314	80F/EO	St. John’s House	3	1690-1715	Tibia	Right	*Rattus* sp.	*R. rattus*	-6.1	5.5	39.8	14.2	3.26	1	Cellar, 80F/EO
HEAL-2315	80F/EP	St. John’s House	3	1690-1715	Tibia	Right	*Rattus* sp.	*R. rattus*	-17.6	7.9	41.7	14.4	3.37	1	Cellar, 80F/EP
TEAL-13383	1B16F7-48	Fortress of Louisbourg	4	No Data	Os Coxa	No Data	*Rattus* sp.	*R. norvegicus*	-19.2	9.7	43.1	15.5	3.24	1	Context 1B16F7, Specimen 48, Box 5
TEAL-13384	1B16G4-608	Fortress of Louisbourg	4	No Data	Os Coxa	No Data	*Rattus* sp.	*R. norvegicus*	-17.6	10.7	43.4	15.7	3.23	1	Context 1B16G4, Specimen 608, Box 7
TEAL-13385	1B16H5-32	Fortress of Louisbourg	4	No Data	Ulna	No Data	*Rattus* sp.	*R. norvegicus*	-18.1	10.9	43.0	15.7	3.22	1	Context 1B16H5, Specimen 32, Box 12
TEAL-13392	1B16C4-441	Fortress of Louisbourg	4	No Data	Tibia	No Data	*Rattus* sp.	*R. norvegicus*	-19.0	9.9	48.0	17.5	3.20	1	Context 1B16C4, Specimen 441, Box 12
TEAL-13393	17L45A4-12	Fortress of Louisbourg	4	1751-1784	Cranium	No Data	*Rattus* sp.	*R. norvegicus*	-20.2	8.1	40.5	14.7	3.21	1	Context 17L45A4, Specimen 12
TEAL-13394	4L19D5-NA	Fortress of Louisbourg	4	1713-1768	Femur	No Data	*Rattus* sp.	*R. norvegicus*	-17.4	12.2	41.0	14.6	3.27	1	Context 4L19D5, Box 45
TEAL-13395	1B16B4-523	Fortress of Louisbourg	4	No Data	Femur	No Data	*Rattus* sp.	*R. norvegicus*	-19.2	10.1	42.1	15.4	3.19	1	Context 1B16B4, Specimen 523, Box 11
TEAL-13396	1B116F4-13	Fortress of Louisbourg	4	No Data	Cranium	Axial	*Rattus* sp.	*R. norvegicus*	-19.4	8.9	42.7	15.6	3.20	1	Context 1B116F4, Specimen 13, Box 10
TEAL-13397	1B16K6-110	Fortress of Louisbourg	4	No Data	Femur	No Data	*Rattus* sp.	*R. norvegicus*	-19.6	8.6	40.4	14.2	3.31	1	Context 1B16K6, Specimen 110, Box 11
TEAL-13398	1B16K6-111	Fortress of Louisbourg	4	No Data	Femur	No Data	*Rattus* sp.	*R. norvegicus*	-20.0	8.4	40.7	14.7	3.23	1	Context 1B16K6, Specimen 111, Box 11

TEAL-13399	1B16K6-112	Fortress of Louisbourg	4	No Data	Femur	No Data	*Rattus* sp.	*R. norvegicus*	-19.4	8.0	42.2	15.5	3.17	1	Context 1B16K6, Specimen 112, Box 11
TEAL-13400	4L52K13-NA	Fortress of Louisbourg	4	1744-1745	Os Coxa	No Data	*Rattus* sp.	*R. norvegicus*	-16.0	14.1	41.1	15.1	3.18	1	Context 4L52K13, Box 47
TEAL-13401	1B16G1-1748	Fortress of Louisbourg	4	No Data	Femur	Left	*Rattus* sp.	*R. norvegicus*	-13.5	9.3	42.1	15.3	3.21	1	Context 1B16G1, Specimen 1748, Box 5
TEAL-13402	1B16G1-1749	Fortress of Louisbourg	4	No Data	Femur	Left	*Rattus* sp.	*R. norvegicus*	-19.2	7.8	43.3	15.6	3.23	1	Context 1B16G1, Specimen 1749, Box 5
TEAL-13403	1B16G1-1752	Fortress of Louisbourg	4	No Data	Femur	Left	*Rattus* sp.	*R. norvegicus*	-19.9	8.5	42.4	15.6	3.18	1	Context 1B16G1, Specimen 1752, Box 5
TEAL-13404	1B16G1-1757	Fortress of Louisbourg	4	No Data	Femur	Right	*Rattus* sp.	*R. norvegicus*	-19.7	8.7	42.2	15.4	3.19	1	Context 1B16G1, Specimen 1757, Box 5
TEAL-13405	1B16G1-1753	Fortress of Louisbourg	4	No Data	Femur	Right	*Rattus* sp.	*R. norvegicus*	-20.1	7.8	43.4	16.0	3.16	1	Context 1B16G1, Specimen 1753, Box 5
TEAL-13406	1B16G1-1745	Fortress of Louisbourg	4	No Data	Femur	Right	*Rattus* sp.	*R. norvegicus*	-20.2	7.7	42.9	15.7	3.20	1	Context 1B16G1, Specimen 1745, Box 5
HEAL-1801	NA	Assemblée nat. Québec	5	c. 1780	Femur	Left	*Rattus* sp.	*R. norvegicus*	-20.8	6.2	43.1	15.6	3.22	1	Lot 2B6, Box 87
HEAL-1814	NA	Assemblée nat. Québec	5	c. 1780	Femur	Right	*Rattus* sp.	*R. norvegicus*	-20.5	7.2	42.5	15.5	3.20	1	Lot 2B7, Box 87
HEAL-1830	NA	Assemblée nat. Québec	5	1740-1760	Humerus	Right	*Rattus* sp.	*R. rattus*	-20.1	5.8	42.1	15.4	3.20	1	Lot 2B11, Box 88
HEAL-1843	NA	rue St.-Vallier	5	1784-1835	Femur	Left	*Rattus* sp.	*R. norvegicus*	-19.8	10.2	40.0	14.5	3.22	1	Lot 2A6, Box 1556
HEAL-1859	NA	rue St.-Vallier	5	1784-1835	Tibia	No Data	*Rattus* sp.	*R. norvegicus*	-21.0	6.2	39.6	14.2	3.25	1	Lot 5F6, Box 1554
HEAL-1868	NA	rue St.-Vallier	5	1784-1835	Humerus	Left	*Rattus* sp.	*R. norvegicus*	-19.4	9.8	39.5	14.3	3.21	1	Lot 5F6 ext., Box 1553
TEAL-3664	1	St. Anne's Market	5	1834-1844	Femur	Right	*Rattus* sp.	*R. norvegicus*	-18.0	12.8	29.4	13.5	2.66	1	Lot 23A22
TEAL-3665	2	St. Anne's Market	5	1834-1844	Femur	Left	*Rattus* sp.	*R. norvegicus*	-19.1	11.7	41.4	12.4	4.06	2	Lot 23A22
TEAL-3666	3	St. Anne's Market	5	1834-1844	Femur	Left	*Rattus* sp.	*R. norvegicus*	-19.6	10.1	40.9	14.1	3.38	1	Lot 23A22
TEAL-3674	11	St. Anne's Market	5	1851-1901	Femur	Right	*Rattus* sp.	*R. norvegicus*	-19.8	10.7	42.7	15.2	3.27	1	Lot 23A5
TEAL-3675	12	St. Anne's Market	5	1851-1901	Femur	Left	*Rattus* sp.	*R. norvegicus*	-19.2	9.8	43.0	15.2	3.30	1	Lot 23A5
TEAL-3676	13	St. Anne's Market	5	1851-1901	Femur	Left	*Rattus* sp.	*R. norvegicus*	-17.8	10.9	38.6	13.6	3.31	1	Lot 23A5
TEAL-3677	14	St. Anne's Market	5	1851-1901	Femur	Right	*Rattus* sp.	*R. norvegicus*	-19.6	10.4	34.9	12.0	3.40	1	Lot 23A5
TEAL-3678	15	St. Anne's Market	5	1851-1901	Femur	Left	*Rattus* sp.	*R. norvegicus*							Lot 23A5
TEAL-3679	16	St. Anne's Market	5	1851-1901	Femur	No Data	*Rattus* sp.	*R. norvegicus*	-18.6	10.5	39.9	14.3	3.27	1	Lot 23A5
TEAL-3680	17	St. Anne's Market	5	1851-1901	Femur	No Data	*Rattus* sp.	*R. norvegicus*	-17.5	10.8	40.5	14.6	3.23	1	Lot 23A5
TEAL-3682	19	St. Anne's Market	5	1851-1901	Femur	No Data	*Rattus* sp.	*R. norvegicus*	-19.8	8.7	30.7	10.8	3.31	1	Lot 23A5
TEAL-3685	22	St. Anne's Market	5	1851-1901	Femur	No Data	*Rattus* sp.	*R. norvegicus*	-18.3	11.9	37.5	14.6	3.01	1	Lot 23A5
TEAL-3691	28	St. Anne's Market	5	1851-1901	Femur	No Data	*Rattus* sp.	*R. norvegicus*	-19.7	9.8	44.3	14.4	3.63	1	Lot 23B22
TEAL-3692	29	St. Anne's Market	5	1851-1901	Femur	No Data	*Rattus* sp.	*R. norvegicus*	-19.9	10.6	42.2	15.9	3.10	1	Lot 23B22
TEAL-3703	40	St. Anne's Market	5	1851-1901	Femur	No Data	*Rattus* sp.	*R. norvegicus*	-18.9	10.1	43.2	15.3	3.29	1	Lot 23B4
TEAL-3704	41	St. Anne's Market	5	1851-1901	Femur	No Data	*Rattus* sp.	*R. norvegicus*	-13.9	11.5	42.5	14.9	3.33	1	Lot 23B4

TEAL-3705	42	St. Anne's Market	5	1851-1901	Femur	No Data	*Rattus* sp.	*R. norvegicus*	-18.7	10.5	41.1	14.9	3.22	1	Lot 23B4
TEAL-3706	43	St. Anne's Market	5	1851-1901	Femur	No Data	*Rattus* sp.	*R. norvegicus*	-19.7	10.5	42.5	15.0	3.30	1	Lot 23B4
TEAL-3707	44	St. Anne's Market	5	1851-1901	Femur	No Data	*Rattus* sp.	*R. norvegicus*	-17.4	11.3	42.6	15.6	3.18	1	Lot 23B4
TEAL-3710	47	St. Anne's Market	5	1851-1901	Femur	No Data	*Mus musculus*	*R. norvegicus*	-18.1	11.1	40.6	14.6	3.25	1	Lot 23B4
TEAL-3724	61	St. Anne's Market	5	1834-1844	Femur	No Data	*Rattus* sp.	*R. norvegicus*	-18.8	8.8	42.4	15.4	3.21	1	Lot 23C17
TEAL-3735	72	St. Anne's Market	5	1851-1901	Femur	No Data	*Rattus* sp.	*R. norvegicus*	-18.8	10.9	36.5	13.2	3.22	1	Lot 23C2
TEAL-3736	73	St. Anne's Market	5	1851-1901	Femur	No Data	*Rattus* sp.	*R. norvegicus*	-19.3	10.4	42.2	15.0	3.28	1	Lot 23C2
TEAL-3737	74	St. Anne's Market	5	1851-1901	Femur	No Data	*Rattus* sp.	*R. norvegicus*	-19.1	10.3	42.3	14.9	3.30	1	Lot 23C2
TEAL-3738	75	St. Anne's Market	5	1851-1901	Femur	No Data	*Rattus* sp.	*R. norvegicus*	-20.0	11.0	40.9	14.3	3.33	1	Lot 23C2
TEAL-3739	76	St. Anne's Market	5	1851-1901	Femur	No Data	*Rattus* sp.	*R. norvegicus*	-18.7	10.3	42.4	15.4	3.21	1	Lot 23C2
TEAL-3740	77	St. Anne's Market	5	1851-1901	Femur	No Data	*Rattus* sp.	*R. norvegicus*	-19.5	9.7	41.6	14.7	3.29	1	Lot 23C2
TEAL-3741	78	St. Anne's Market	5	1851-1901	Femur	No Data	*Rattus* sp.	*R. norvegicus*	-18.6	11.2	41.1	14.9	3.22	1	Lot 23C2
TEAL-3742	79	St. Anne's Market	5	1851-1901	Femur	No Data	*Rattus* sp.	*R. norvegicus*	-19.7	10.4	42.3	15.3	3.24	1	Lot 23C2
TEAL-3743	80	St. Anne's Market	5	1851-1901	Femur	No Data	*Rattus* sp.	*R. norvegicus*	-20.5	10.5	40.1	14.0	3.35	1	Lot 23C2
TEAL-3753	90	St. Anne's Market	5	1851-1901	Femur	No Data	*Rattus* sp.	*R. norvegicus*	-18.1	11.1	40.9	14.9	3.21	1	Lot 23D2
TEAL-3754	91	St. Anne's Market	5	1851-1901	Femur	No Data	*Rattus* sp.	*R. norvegicus*	-18.0	11.2	40.0	14.5	3.23	1	Lot 23D2
TEAL-3755	92	St. Anne's Market	5	1851-1901	Femur	No Data	*Rattus* sp.	*R. norvegicus*	-19.1	10.7	41.2	14.9	3.23	1	Lot 23D2
TEAL-3756	93	St. Anne's Market	5	1851-1901	Femur	No Data	*Rattus* sp.	*R. norvegicus*	-18.9	11.2	41.7	14.8	3.28	1	Lot 23D2
TEAL-3757	94	St. Anne's Market	5	1851-1901	Femur	No Data	*Rattus* sp.	*R. norvegicus*	-19.3	9.2	47.2	17.0	3.24	1	Lot 23D2
TEAL-3763	100	St. Anne's Market	5	1834-1844	Femur	No Data	*Rattus* sp.	*R. norvegicus*	-20.0	10.5	41.0	14.8	3.22	1	Lot 23A27
TEAL-3770	107	St. Anne's Market	5	1851-1901	Tibia	No Data	*Rattus* sp.	*R. norvegicus*	-17.7	10.4	41.6	14.9	3.25	1	Lot 23C3
TEAL-3771	108	St. Anne's Market	5	1851-1901	Tibia	No Data	*Rattus* sp.	*R. norvegicus*	-12.0	7.8	39.2	13.8	3.32	1	Lot 23C3
TEAL-3772	109	St. Anne's Market	5	1851-1901	Tibia	No Data	*Rattus* sp.	*R. norvegicus*	-16.3	10.3	36.6	12.5	3.41	1	Lot 23C3
TEAL-3776	113	St. Anne's Market	5	1834-1844	Tibia	No Data	*Rattus* sp.	*R. norvegicus*	-20.4	9.3	41.0	14.2	3.36	1	Lot 23A22
TEAL-3777	114	St. Anne's Market	5	1834-1844	Tibia	No Data	*Rattus* sp.	*R. norvegicus*	-20.0	10.8	40.0	14.5	3.21	1	Lot 23A22
TEAL-3778	115	St. Anne's Market	5	1834-1844	Tibia	No Data	*Rattus* sp.	*R. norvegicus*	-18.9	10.1	39.7	14.4	3.21	1	Lot 23A22
TEAL-3779	116	St. Anne's Market	5	1834-1844	Tibia	No Data	*Rattus* sp.	*R. norvegicus*	-18.4	11.7	41.6	15.2	3.20	1	Lot 23A22
TEAL-3780	117	St. Anne's Market	5	1851-1901	Femur	No Data	*Rattus* sp.	*R. norvegicus*	-18.7	10.9	43.0	15.6	3.23	1	Lot 23B4
TEAL-3781	118	St. Anne's Market	5	1851-1901	Femur	No Data	*Rattus* sp.	*R. norvegicus*	-19.7	11.6	42.0	15.3	3.21	1	Lot 23B4
TEAL-3850	187	St. Anne's Market	5	1834-1844	Femur	No Data	*Rattus* sp.	*R. norvegicus*	-19.3	11.5	40.8	14.4	3.31	1	Lot 30G25

TEAL-3868	205	St. Anne's Market	5	1844-1849	Femur	No Data	*Rattus* sp.	*R. norvegicus*	-19.8	10.5	40.4	14.5	3.24	1	Lot 31H47
TEAL-3871	208	St. Anne's Market	5	1834-1844	Femur	No Data	*Rattus* sp.	*R. norvegicus*	-19.8	10.2	42.4	15.3	3.22	1	Lot 32K15
TEAL-3876	213	St. Anne's Market	5	1834-1844	Femur	No Data	*Rattus* sp.	*R. norvegicus*	-19.5	11.0	41.6	14.8	3.28	1	Lot 30G33
TEAL-3882	219	St. Anne's Market	5	1834-1844	Tibia	No Data	*Rattus* sp.	*R. norvegicus*	-19.2	10.9	41.9	15.2	3.23	1	Lot 32J28
TEAL-3883	220	St. Anne's Market	5	1834-1844	Tibia	No Data	*Rattus* sp.	*R. norvegicus*	-19.8	10.8	42.2	14.1	3.52	1	Lot 32J28
TEAL-3884	221	St. Anne's Market	5	1834-1844	Tibia	No Data	*Rattus* sp.	*R. norvegicus*	-18.3	11.1	42.7	15.1	3.30	1	Lot 32J28
TEAL-3903	240	St. Anne's Market	5	1834-1844	Femur	No Data	*Rattus* sp.	*R. norvegicus*	-18.5	11.5	42.5	15.3	3.24	1	Lot 32K29
TEAL-3904	241	St. Anne's Market	5	1834-1844	Femur	No Data	*Rattus* sp.	*R. norvegicus*	-19.9	10.1	42.4	15.5	3.19	1	Lot 32K29
TEAL-3681	18	St. Anne's Market	5	1851-1901	Femur	No Data	*Rattus* sp.	Unsuccessful	-16.6	11.2	39.1	15.0	3.06	1	Lot 23A5
IUBC-2678	NA	Ferryland	6	1623-1628	Femur	Right	*Rattus* sp.	Not sampled	-18.2	8.7	41.2	14.6	3.28	1	Unit E67S6, Event 919, Area F
IUBC-2679	NA	Ferryland	6	1623-1628	Femur	Right	*Rattus* sp.	Not sampled	-17.8	11.2	40.6	14.4	3.28	1	Unit E67S6, Event 919, Area F
IUBC-2680	NA	Ferryland	6	1623-1628	Femur	Right	*Rattus* sp.	Not sampled	-17.8	11.2	39.9	14.1	3.30	1	Unit E67S6, Event 919, Area F
IUBC-2681	NA	Ferryland	6	1623-1628	Femur	Right	*Rattus* sp.	Not sampled	-18.9	8.8	40.3	14.2	3.31	1	Unit E67S6, Event 919, Area F
SUBC-1725	CgAf-2:209034	Ferryland	6	1700s-1900s	Tibia	No Data	*Rattus* sp.	*R. norvegicus*	-18.0	15.1	41.5	14.1	3.43	1	Event 306, mixed 'plough zone' deposit in Area G.
SUBC-1730	NA	Ferryland	6	1622-1696	Humerus	No Data	*Rattus* sp.	*R. norvegicus*	-15.5	16.2	41.5	14.9	3.25	1	Unit E56N4, Event 644
SUBC-1732	NA	Ferryland	6	1700s	Humerus	No Data	*Rattus* sp.	*R. norvegicus*	-17.1	12.5	43.6	15.4	3.30	1	Event 847, inside Structure 19
SUBC-1736	NA	Ferryland	6	1697-1700s	Mandible	No Data	*Rattus* sp.	*R. norvegicus*	-14.6	19.3	42.2	14.5	3.40	1	Unit S13E54, Event 873
SUBC-1737	NA	Ferryland	6	1697-1700s	Mandible	No Data	*Rattus* sp.	*R. norvegicus*	-15.1	18.1	42.3	14.8	3.33	1	Unit S13E54, Event 873
SUBC-1738	NA	Ferryland	6	1697-1700s	Mandible	No Data	*Rattus* sp.	*R. norvegicus*	-17.4	11.7	41.5	14.8	3.28	1	Unit S13E54, Event 873
SUBC-1739	NA	Ferryland	6	1697-1700s	Mandible	No Data	*Rattus* sp.	*R. norvegicus*	-16.5	16.4	38.2	13.5	3.29	1	Unit E53S14, Event 873
SUBC-1740	NA	Ferryland	6	1697-1700s	Mandible	No Data	*Rattus* sp.	*R. norvegicus*	-17.5	12.5	42.2	15.1	3.26	1	Unit E53S14, Event 873
MARC-325	NA	Ferryland	6	1622-1696	Femur	Left	*Rattus* sp.	*R. rattus*	-19.2	7.6	43.9	15.6	3.28	1	Event 634
MARC-326	NA	Ferryland	6	1622-1696	Femur	Left	*Rattus* sp.	*R. rattus*	-19.2	7.1	43.8	15.7	3.25	1	Event 634
MARC-327	NA	Ferryland	6	1622-1696	Femur	Left	*Rattus* sp.	*R. rattus*	-18.6	6.4	44.3	15.8	3.28	1	Event 634
MARC-328	NA	Ferryland	6	1622-1696	Femur	Left	*Rattus* sp.	*R. rattus*	-18.0	7.9	44.7	16.0	3.27	1	Event 634
SUBC-1710	478347	Ferryland	6	1640-1650s	Tibia	No Data	*Mus musculus*	*R. rattus*	-21.9	7.5	39.2	10.7	4.28	2	Unit E067N003, Event 613
SUBC-1711	NA	Ferryland	6	1622-1696	Tibia	No Data	*Rattus* sp.	*R. rattus*	-18.2	14.2	40.0	13.0	3.59	1	Unit E069S004, Event 651
SUBC-1712	NA	Ferryland	6	1622-1696	Tibia	No Data	*Rattus* sp.	*R. rattus*	-17.1	13.0	42.2	14.9	3.30	1	Unit E069S004, Event 651
SUBC-1713	490364	Ferryland	6	1622-1696	Humerus	No Data	*Rattus* sp.	*R. rattus*	-18.3	12.5	41.4	13.8	3.50	1	Unit E065S003, Event 627
SUBC-1714	NA	Ferryland	6	1622-1696	Femur	No Data	*Rattus* sp.	*R. rattus*	-16.8	14.1	42.2	14.8	3.33	1	Unit E056S007, Event 634

SUBC-1715	NA	Ferryland	6	1622-1696	Femur	No Data	*Rattus* sp.	*R. rattus*	-19.0	7.4	41.6	14.7	3.31	1	Unit E057S006, Event 634
SUBC-1726	NA	Ferryland	6	1622-1800	Femur	No Data	*Rattus* sp.	*R. rattus*	-17.7	13.0	40.9	14.0	3.40	1	Unit E73-70S4, Event 0
SUBC-1727	NA	Ferryland	6	1630s	Femur	No Data	*Rattus* sp.	*R. rattus*	-19.5	7.8	41.6	14.7	3.31	1	Event 116
SUBC-1729	NA	Ferryland	6	1750s-1790s	Humerus	No Data	*Rattus* sp.	*R. rattus*	-18.0	11.2	41.5	14.8	3.28	1	Unit E41S5, Event 694, Area F
SUBC-1734	NA	Ferryland	6	1622-1696	Mandible	No Data	*Rattus* sp.	*R. rattus*	-21.5	3.1	43.3	15.0	3.36	1	Unit E52S9, Event 634
SUBC-1741	NA	Ferryland	6	1622-1696	Tibia	No Data	*Rattus* sp.	*R. rattus*	-18.5	11.7	42.7	15.4	3.23	1	Unit E066N001, Event 627
SUBC-1716	NA	Ferryland	6	1622-1696	Femur	No Data	*Rattus* sp.	Unsuccessful	-19.0	9.0	41.6	14.4	3.36	1	Unit E056S005, Event 634
SUBC-1717	NA	Ferryland	6	1622-1696	Femur	No Data	*Rattus* sp.	Unsuccessful	-20.4	8.6	40.5	12.4	3.81	1	Unit E056S005, Event 634
SUBC-1718	490373	Ferryland	6	1622-1696	Humerus	No Data	*Rattus* sp.	Unsuccessful	-18.8	11.9	40.9	13.4	3.57	1	Unit E065S003, Event 627
SUBC-1719	42849	Ferryland	6	c. 1673	Innominate	No Data	*Rattus* sp.	Unsuccessful	-19.4	11.5	41.4	14.0	3.45	1	Event 49
SUBC-1720	76442	Ferryland	6	1650-1673	Tibia	No Data	*Rattus* sp.	Unsuccessful	-16.4	15.5	41.3	12.8	3.75	2	Event 50
SUBC-1721	76041	Ferryland	6	1640s	Maxilla	No Data	*Rattus* sp.	Unsuccessful	-19.0	10.0	41.2	14.0	3.43	1	Event 111
SUBC-1722	399187	Ferryland	6	1650-1696	Maxilla	No Data	*Rattus* sp.	Unsuccessful	-18.5	12.4	40.7	13.8	3.44	1	Event 509
SUBC-1723	CgAf-2:83799	Ferryland	6	1622-1673	Innominate	No Data	*Rattus* sp.	Unsuccessful	-19.6	11.4	41.2	13.4	3.58	1	Excavation cat no. CgAf-2:83799
SUBC-1724	CgAf-2:31351	Ferryland	6	1650-1673	Femur	No Data	*Rattus* sp.	Unsuccessful	-17.4	14.1	41.5	14.5	3.35	1	Event 44
SUBC-1728	NA	Ferryland	6	1622-1696	Tibia	No Data	*Rattus* sp.	Unsuccessful	-18.7	9.5	41.5	14.5	3.34	1	Unit E70S1, Event 481
SUBC-1731	NA	Ferryland	6	1700s	Humerus	No Data	*Rattus* sp.	Unsuccessful	-18.5	14.6	42.0	13.9	3.52	1	Event 847, inside Structure 19
SUBC-1733	NA	Ferryland	6	1697-1720s	Ulna	No Data	*Rattus* sp.	Unsuccessful	-16.5	17.5	41.9	13.8	3.54	1	Event 637, Area F.
SUBC-1735	NA	Ferryland	6	1622-1696	Mandible	No Data	*Rattus* sp. or *M. musculus*	Unsuccessful	-22.2	3.3	42.1	13.6	3.61	1	Unit E52S10, Event 634
HEAL-21a	269.051	Emanuel Point Wreck 1	7	Sank 1559	Femur	Right	*R. rattus*	*R. rattus*	-19.0	8.4	39.7	13.9	3.34	1	Project 94L, Site 8ES1980, Grid 114N127E, Level 3
HEAL-21b	269.051	Emanuel Point Wreck 1	7	Sank 1559	Femur	Right	*R. rattus*	*R. rattus*	-18.5	9.0	40.2	14.3	3.28	1	Project 94L, Site 8ES1980, Grid 114N127E, Level 3
HEAL-22	602.001	Emanuel Point Wreck 1	7	Sank 1559	Femur	Right	*R. rattus*	*R. rattus*	-17.3	8.5	40.9	14.4	3.32	1	Project 95L, Site 8ES1980
HEAL-12	2059.007	Emanuel Point Wreck 2	7	Sank 1559	Femur	Left	*R. rattus*	*Mus* sp.	-15.6	7.5	28.9	10.0	3.38	1	Project 09W, Site 8ES3345, Grid 85N499E, Depth below datum 65-90cm
HEAL-15	2003.001	Emanuel Point Wreck 2	7	Sank 1559	Femur	Left	*Rattus* sp.	*Mus* sp.	-18.3	7.4	33.5	11.8	3.33	1	Project 09W, Site 8ES3345, Grid 85N499E, Depth below datum 40-60cm, INFANT
HEAL-11	1485.001	Emanuel Point Wreck 2	7	Sank 1559	Femur	Left	*R. rattus*	*R. rattus*	-12.2	7.7	35.3	10.4	3.97	2	Project 08W, Site 8ES3345, Grid 86N497E, Depth below datum 60-80cm
HEAL-13	2059.012	Emanuel Point Wreck 2	7	Sank 1559	Femur	Left	*Rattus* sp.	*R. rattus*	-18.9	6.6	34.9	11.7	3.48	1	Project 09W, Site 8ES3345, Grid 85N499E, Depth below datum 65-90cm
HEAL-14	2090.002	Emanuel Point Wreck 2	7	Sank 1559	Femur	Left	*Rattus* sp.	*R. rattus*	-16.9	10.2	41.6	15.0	3.24	1	Project 09W, Site 8ES3345, Grid 93N490E, Depth below datum 40-60
IUBC-5506	2476	*La Belle*	7	1684-1686	Femur	Right	*R. rattus*	*R. rattus*	-16.7	9.1	43.3	15.1	3.34	1	Unit 2007E2008N, Context: Southwest of Ship
IUBC-5507	3037	*La Belle*	7	1684-1686	Femur	Right	*R. rattus*	*R. rattus*	-15.9	9.4	44.5	15.2	3.41	1	Unit 2011E2019N, Context: Bow

IUBC-5509	5938	*La Belle*	7	1684-1686	Mandible	Left	*R. rattus*	*R. rattus*	-16.0	9.1	42.9	14.5	3.45	1	Unit 2011E2014N, Context: Main Hold
IUBC-5510	6204	*La Belle*	7	1684-1686	Femur	Left	*R. rattus*	*R. rattus*	-15.6	8.8	42.9	15.1	3.31	1	Unit 2010E2020N, Context: Bow
IUBC-5512	10403	*La Belle*	7	1684-1686	Femur	Left	*R. rattus*	*R. rattus*	-17.6	9.6	43.3	14.5	3.49	1	Unit 2010E2011N, Context: Aft Hold
IUBC-5515	4909-4	*La Belle*	7	1684-1686	Humerus	No Data	*R. rattus*	*R. rattus*	-16.1	8.8	42.5	14.2	3.49	1	Context: Bow, found during conservation of the rope
IUBC-5508	5211	*La Belle*	7	1684-1686	Femur	Right	*R. rattus*	Unsuccessful	-16.1	9.0	43.3	14.7	3.42	1	Unit 2011E2006N, Context: Stern
IUBC-5511	6328	*La Belle*	7	1684-1686	Femur	Right	*R. rattus*	Unsuccessful	-17.1	9.1	43.5	14.8	3.43	1	Unit 2009E2006N, Context: Stern
IUBC-5513	10477	*La Belle*	7	1684-1686	Mandible	Left	*R. rattus*	Unsuccessful	-16.4	9.9	43.0	14.5	3.46	1	Unit 2011E2014N
IUBC-5514	10699.1	*La Belle*	7	1684-1686	Femur	Right	*R. rattus*	Unsuccessful	-16.3	8.7	41.6	14.1	3.44	1	Unit 2010E2011N, Context: Aft Hold
IUBC-2583	26	*Le Machault*	7	1757-1760	Innominate	Right	*Rattus* sp.	*R. norvegicus*	-16.6	10.3	41.5	14.5	3.33	1	INV Rec no. 1172, Prov no. 2M40A1, Operation 40, Lot (none listed), "B"
IUBC-2584	27	*Le Machault*	7	1757-1760	Innominate	Right	*Rattus* sp.	Unsuccessful	-18.5	7.6	41.5	14.2	3.40	1	INV Rec no. 1169, Prov no. 2M38D1, Operation 38, Lot 1, "B"
HEAL-3293	3587.003	*Queen Anne's Revenge*	7	1710-1718	Vertebra	Axial	*Rattus* sp.	*R. rattus*							3587.003, VOA CAB5 07/17/17
HEAL-3292	0387.003	*Queen Anne's Revenge*	7	1710-1718	Ulna	No Data	*Rattus* sp.	Unsuccessful							0387.003, NCMM 05/27/04
HEAL-32	408.001	*Rosario*	7	1696-1705	Humerus	Left	*R. rattus*	*R. rattus*	-15.0	8.1	41.2	14.7	3.27	1	Project 01SRI, Site 8ES1905, Grid 124N418E
HEAL-36	25.001	*Rosario*	7	1696-1705	Tibia	Right	*R. rattus*	*R. rattus*	-17.6	6.1	41.2	14.6	3.28	1	Project 02SRI, Site 8ES1905, Grid 120N416E, Quad SW, Depth below datum 80cm
HEAL-37	249.001	*Rosario*	7	1696-1705	Tibia	Left	*R. rattus*	*R. rattus*	-18.2	6.2	41.7	14.9	3.26	1	Project 02SRI, Site 8ES1905, Grid 120N420E, Quad NW, Depth below datum 140cm
HEAL-38	406.001	*Rosario*	7	1696-1705	Innominate	Left	*R. rattus*	*R. rattus*	-17.9	7.1	41.1	14.6	3.29	1	Project 02SRI, Site 8ES1905, Grid 120N418E, Quad NW
HEAL-39	408.001	*Rosario*	7	1696-1705	Tibia	Right	*R. rattus*	*R. rattus*	-18.2	6.2	41.9	15.0	3.26	1	Project 02SRI, Site 8ES1905, Grid 120N418E, Quad NW
HEAL-40	409.001	*Rosario*	7	1696-1705	Humerus	Left	*R. rattus*	*R. rattus*	-17.5	6.6	40.8	14.4	3.31	1	Project 02SRI, Site 8ES1905, Grid 120N418E, Quad NW
HEAL-58	402.001	*Rosario*	7	1696-1705	Tibia	Left	*R. rattus*	*R. rattus*	-17.1	6.7	41.9	15.1	3.25	1	Project 99SRI, Site 8ES1905, Grid 120N420E, Quad NE, Depth below datum 105cm
HEAL-59	490.001	*Rosario*	7	1696-1705	Innominate	Left	*R. rattus*	*R. rattus*	-15.3	8.4	41.4	14.6	3.30	1	Project 99SRI, Site 8ES1905, Grid 120N422E, Quad NW, Depth below datum 95cm
HEAL-60	493.001	*Rosario*	7	1696-1705	Humerus	Left	*R. rattus*	*R. rattus*	-17.4	6.6	41.3	14.7	3.28	1	Project 99SRI, Site 8ES1905, Grid 120N422E, Quad NW, Depth below datum 95cm
HEAL-61	740.001	*Rosario*	7	1696-1705	Innominate	Left	*R. rattus*	*R. rattus*	-16.9	7.7	40.9	14.6	3.27	1	Project 99SRI, Site 8ES1905, Grid 122N420E, Quad SW, Depth below datum 85cm
HEAL-2306	24M8N10-4	*San Jaun*	7	Sank 1565	Humerus	Right	*Rattus* sp. cf	*R. rattus*	-18.9	12.4	40.0	13.9	3.36	1	Sank 1565

Table S3. Means and standard deviations (1σ) for ZooMS-identified black and brown rats at all sites. Asterisk indicates that the site was connected with a fishing or whaling station (which are not included in our discussion of interspecific trophic level differences).

Site Name	Taxa	Region	*n*=	δ^{13}C (‰, 1σ)	δ^{15}N (‰, 1σ)
1231 Bourbon St.	*R. norvegicus*	New Orleans	4	-16.1 ± 3.1	8.9 ± 0.9
626 Bourbon St.	*R. norvegicus*	New Orleans	8	-14.1 ± 2.0	9.8 ± 1.2
810 Royal St.	*R. norvegicus*	New Orleans	11	-14.7 ± 2.2	10.2 ± 1.5
936 St. Peter St.	*R. norvegicus*	New Orleans	14	-14.2 ± 0.8	10.4 ± 0.8
936 St. Peter St.	*R. rattus*	New Orleans	1	-17.6	9.3
Passebon Cottage	*R. norvegicus*	New Orleans	4	-12.9 ± 1.5	10.1 ± 1.6
Rising Sun	*R. norvegicus*	New Orleans	9	-16.5 ± 2.1	10.0 ± 0.8
St. Anthony's Garden	*R. norvegicus*	New Orleans	3	-13.2 ± 3.6	9.3 ± 0.7
Ursuline Convent	*R. norvegicus*	New Orleans	9	-15.5 ± 2.3	9.9 ± 0.9
New Orleans (all)	*R. norvegicus*	New Orleans	62	-14.8 ± 2.2	10.0 ± 1.1
New Orleans (all)	*R. rattus*	New Orleans	1	-17.6	9.3
Atlantic Wharf	*R. norvegicus*	Charleston	2	-11.5 ± 0.9	7.5 ± 2.1
Heyward Washington	*R. norvegicus*	Charleston	2	-11.6 ± 4.1	8.6 ±
Heyward Washington	*R. rattus*	Charleston	3	-15.5 ± 3.3	8.7 ±
South Adgers Wharf	*R. norvegicus*	Charleston	4	-15.3 ± 3.2	8.6 ± 1.2
South Adgers Wharf	*R. rattus*	Charleston	1	-13.2	7.6 0.0
Charleston (all)	*R. norvegicus*	Charleston	8	-13.4 ± 3.3	8.3 ± 1.2
Charleston (all)	*R. rattus*	Charleston	4	-14.9 ± 2.9	8.4 ± 0.7
Hotel Indigo	*R. norvegicus*	Chesapeake	10	-13.4 ± 3.3	10.7 ± 1.8
House for Families	*R. rattus*	Chesapeake	12	-11.2 ± 3.0	8.9 ± 1.2
Jamestown	*R. rattus*	Chesapeake	10	-16.0 ± 2.6	8.3 ± 0.5
Madison Basement	*R. norvegicus*	Chesapeake	3	-12.7 ± 2.8	8.0 ± 0.6
Madison Basement	*R. rattus*	Chesapeake	1	-6.2	6.7
Mount Pleasant	*R. rattus*	Chesapeake	5	-13.1 ± 1.1	7.8 ± 0.6
Richneck Quarter	*R. rattus*	Chesapeake	7	-11.5 ± 4.7	8.6 ± 1.1

South Grove	*R. norvegicus*	Chesapeake	1	-8.3	10.2
South Grove	*R. rattus*	Chesapeake	1	-8.2	7.8
St. John's House	*R. rattus*	Chesapeake	9	-11.9 ± 4.9	7.8 ± 2.2
Chesapeake (all)	*R. norvegicus*	Chesapeake	14	-12.9 ± 3.2	10.1 ± 1.9
Chesapeake (all)	*R. rattus*	Chesapeake	45	-12.5 ± 4.0	8.3 ± 1.3
Assemblée nationale du Québec	*R. norvegicus*	Quebec	2	-20.7 ± 0.2	6.7 ± 0.7
Assemblée nationale du Québec	*R. rattus*	Quebec	1	-20.1	5.8
rue St.-Vallier	*R. norvegicus*	Quebec	3	-20.1 ± 0.8	8.7 ± 2.2
St. Anne Market	*R. norvegicus*	Quebec	52	-18.8 ± 1.5	10.6 ± 0.8
Quebec (all)	*R. norvegicus*	Quebec	57	-18.9 ± 1.5	10.3 ± 1.2
Quebec (all)	*R. rattus*	Quebec	1	-20.1	5.8
Emanuel Point Wreck 1	*R. rattus*	Gulf of Mexico	3	-18.3 ± 0.9	8.6 ± 0.3
Emanuel Point Wreck 2	*R. rattus*	Gulf of Mexico	2	-17.9 ± 1.4	8.4 ± 2.5
La Belle	*R. rattus*	Gulf of Mexico	6	-16.3 ± 0.7	9.1 ± 0.3
Rosario	*R. rattus*	Gulf of Mexico	10	-17.1 ± 1.1	7.0 ± 0.8
Ferryland*	*R. norvegicus*	Maritimes/Newfoundland	8	-16.5 ± 1.3	15.2 ± 2.8
Ferryland*	*R. rattus*	Maritimes/Newfoundland	15	-18.8 ± 1.4	9.6 ± 3.4
Fortress of Louisbourg	*R. norvegicus*	Maritimes/Newfoundland	18	-18.8 ± 1.7	9.4 ± 1.7
*San Jaun**	*R. rattus*	Maritimes/Newfoundland	1	-18.9	12.4
Le Machault	*R. norvegicus*	Maritimes/Newfoundland	1	-16.6	10.3
Rats on ships	*R. rattus*	NA	22	-17.2 ± 1.2	8.2 ± 1.6
Rats not on ships	*R. rattus*	NA	48	-13.9 ± 4.0	8.3 ± 1.3
All (non fishing station)	*R. norvegicus*	NA	159	-16.5 ± 3.1	10.0 ± 1.4
All (non fishing station)	*R. rattus*	NA	72	-14.2 ± 4.0	8.2 ± 1.3

Table S4. Means and standard deviations (1σ) for ZooMS-identified black and brown rats at sites where both are present. Asterisk indicates that the site was a fishing station (which is not included in our discussion of interspecific trophic level differences).

Site	Taxa	*n*=	δ^{13}C (‰, 1σ)	δ^{15}N (‰, 1σ)
936 St. Peter St.	*R. norvegicus*	14	-14.2 ± 0.8	10.4 ± 0.8
	R. rattus	1	-17.6	9.3
	Difference		***3.4***	***1.1***
Heyward Washington	*R. norvegicus*	2	-11.6 ± 4.1	8.6 ±
	R. rattus	3	-15.5 ± 3.3	8.7 ±
	Difference		***3.9***	***-0.1***
South Adgers Wharf	*R. norvegicus*	4	-15.3 ± 3.2	8.6 ± 1.2
	R. rattus	1	-13.2	7.6 0.0
	Difference		***-2.1***	***1.0***
Madison Basement	*R. norvegicus*	3	-12.7 ± 2.8	8.0 ± 0.6
	R. rattus	1	-6.2	6.7
	Difference		***-6.5***	***1.3***
South Grove	*R. norvegicus*	1	-8.3	10.2
	R. rattus	1	-8.2	7.8
	Difference		***-0.1***	***2.4***
Assemblée nationale du Québec	*R. norvegicus*	2	-20.7 ± 0.2	6.7 ± 0.7
	R. rattus	1	-20.1	5.8
	Difference		***-0.5***	***0.9***
Ferryland (*fishing station site)	*R. norvegicus*	8	-16.5 ± 1.3	15.2 ± 2.8
	R. rattus	15	-18.8 ± 1.4	9.6 ± 3.4
	Difference		***2.3***	***5.6***
Chesapeake (all sites)	*R. norvegicus*	14	-12.9 ± 3.2	10.1 ± 1.9
	R. rattus	45	-12.5 ± 4.0	8.3 ± 1.3
	Difference		***-0.4***	***1.8***
All non fishing station sites	*R. norvegicus*	159	-16.5 ± 3.1	10.0 ± 1.4
	R. rattus	72	-14.2 ± 4.0	8.2 ± 1.3

	Difference	***-2.3***	***1.8***

Table S5. Accepted (calibration) and observed long-term (check) isotopic compositions and standard deviations (1σ) for standards used in this study (*91-93*).

Name	Material	Number	δ^{13}C (‰, VPDB)	δ^{15}N (‰, AIR)	Standard Type
USGS40	Glutamic acid	NA	−26.39±0.04	−4.52±0.06	Calibration standard
USGS41a	Glutamic acid	NA	+36.55±0.08	+47.55±0.15	Calibration standard
USGS63	Glutamic acid	NA	−1.17±0.04	+37.83±0.06	Calibration standard
USGS66	Glutamic acid	NA	−0.67±0.04	+40.83±0.06	Calibration standard
MET	Methionine	1608	−28.61±0.10	−5.04±0.13	Check standard
SRM-1	Caribou bone collagen	837	−19.39±0.09	+1.85±0.19	Check standard
SRM-2	Walrus bone collagen	369	−14.80±0.07	+15.58±0.18	Check standard
SRM-3	Commercial gelatine	94	−15.30±0.17	+5.09±0.15	Check standard
SRM-14	Polar bear bone collagen	907	−13.63±0.09	+21.56±0.23	Check standard
SRM-15	Deer bone collagen	329	−26.89±0.08	+6.91±0.18	Check standard
SRM-16	Seal bone collagen	273	−14.78±0.08	+16.93±0.19	Check standard
SRM-25	Caffeine	111	−33.24±0.08	−12.85±0.31	Check standard
SRM-26	GH marine collagen	786	−16.17±0.10	+14.69±0.18	Check standard
SRM-28	Alanine	353	−16.27±0.09	−1.94±0.18	Check standard

Table S6. Means and standard deviations (1σ) for calibration standards for all analytical sessions.

Standard	Analytical Session	Number	$\delta^{13}C$ (1σ)	$\delta^{15}N$ (1σ)
USGS40	CN17-08	10	-26.39 ± 0.05	-4.52 ± 0.12
USGS40	CN17-16	4	-26.39 ± 0.03	-4.52 ± 0.08
USGS40	CN18-10	8	-26.39 ± 0.40	-4.52 ± 0.04
USGS40	CN19-02	5	-26.39 ± 0.09	-4.52 ± 0.18
USGS40	CN19-07	8	-26.39 ± 0.03	-4.52 ± 0.18
USGS40	CN19-09	9	-26.39 ± 0.05	-4.52 ± 0.12
USGS40	CN19-10	9	-26.39 ± 0.05	-4.52 ± 0.18
USGS40	CN19-11	9	-26.39 ± 0.03	-4.52 ± 0.17
USGS40	CN19-12	9	-26.39 ± 0.02	-4.52 ± 0.28
USGS40	CN19-20	9	-26.39 ± 0.03	-4.52 ± 0.20
USGS40	CN19-21	7	-26.39 ± 0.04	-4.52 ± 0.12
USGS40	CN19-22	8	-26.39 ± 0.04	-4.52 ± 0.09
USGS40	CN19-24	9	-26.39 ± 0.02	-4.52 ± 0.25
USGS40	CN20-05	10	-26.39 ± 0.03	-4.52 ± 0.12
USGS40	CN20-10	6	-26.39 ± 0.16	-4.52 ± 0.29
USGS40	CN20-13	6	-26.39 ± 0.13	-4.52 ± 0.21
USGS40	CN21-01	6	-26.39 ± 0.02	-4.52 ± 0.54
USGS40	CN21-02	8	-26.39 ± 0.03	-4.52 ± 0.30
USGS40	CN21-03	6	-26.39 ± 0.03	-4.52 ± 0.10
USGS40	CN22-01	6	-26.39 ± 0.03	-4.61 ± 0.32
USGS40	CN22-10	3	-26.39 ± 0.06	-4.52 ± 0.32
USGS40	CN22-13	6	-26.39 ± 0.04	-4.52 ± 0.34
USGS40	CN22-14	6	-26.39 ± 0.03	-4.44 ± 0.18
USGS40	CN22-20	3	-26.39 ± 0.04	-4.34 ± 0.35
USGS40	CN22-23	5	-26.34 ± 0.07	-4.66 ± 0.47
USGS40	CN23-05	6	-26.25 ± 0.09	-4.44 ± 0.11
USGS40	CN23-07	6	-26.39 ± 0.08	-4.53 ± 0.64
USGS40	CN23-08	6	-26.35 ± 0.08	-4.44 ± 0.22
USGS41a	CN17-08	10	36.55 ± 0.08	47.55 ± 0.37
USGS41a	CN17-16	2	36.55 ± 0.03	47.55 ± 0.25
USGS41a	CN18-10	8	36.55 ± 0.25	47.55 ± 0.14
USGS41a	CN19-02	5	36.55 ± 0.03	47.55 ± 0.13
USGS41a	CN19-07	5	36.55 ± 0.07	47.55 ± 0.27
USGS41a	CN19-09	7	36.55 ± 0.03	47.55 ± 0.30
USGS41a	CN19-10	7	36.55 ± 0.07	47.55 ± 0.25
USGS41a	CN19-11	8	36.55 ± 0.05	47.55 ± 0.25
USGS41a	CN19-12	8	36.55 ± 0.06	47.55 ± 0.34
USGS41a	CN19-20	9	36.55 ± 0.04	47.55 ± 0.26

USGS41a	CN19-21	6	36.55	±	0.08	47.55	±	0.26
USGS41a	CN19-22	8	36.55	±	0.05	47.55	±	0.25
USGS41a	CN19-24	5	36.55	±	0.02	47.55	±	0.18
USGS41a	CN20-05	9	36.55	±	0.04	47.55	±	0.26
USGS41a	CN20-10	4	36.55	±	0.15	47.55	±	0.28
USGS41a	CN20-13	4	36.55	±	0.03	47.55	±	0.40
USGS63	CN21-01	6	-1.17	±	0.07	37.83	±	0.61
USGS63	CN21-02	6	-1.17	±	0.05	37.83	±	0.11
USGS63	CN21-03	6	-1.17	±	0.26	37.83	±	0.16
USGS63	CN22-01	6	-1.17	±	0.08	37.86	±	0.35
USGS63	CN22-10	4	-1.17	±	0.03	37.83	±	0.20
USGS63	CN22-14	5	-1.18	±	0.06	37.91	±	0.12
USGS63	CN22-20	3	-1.17	±	0.05	37.81	±	0.27
USGS63	CN22-23	6	-1.16	±	0.18	37.66	±	0.43
USGS63	CN23-05	6	-1.09	±	0.15	37.97	±	0.20
USGS63	CN23-07	6	-1.07	±	0.17	37.89	±	0.52
USGS63	CN23-08	6	-1.13	±	0.12	37.91	±	0.14
USGS66	CN22-01	6	-0.71	±	0.10	41.25	±	0.67
USGS66	CN22-13	6	-0.67	±	0.13	40.83	±	0.25
USGS66	CN22-14	6	-0.67	±	0.13	40.89	±	0.20
USGS66	CN22-20	2	-0.66	±	0.05	41.17	±	0.29
USGS66	CN22-23	6	-0.66	±	0.07	40.94	±	0.25
USGS66	CN23-05	6	-0.69	±	0.13	40.83	±	0.17
USGS66	CN23-07	6	-0.67	±	0.18	40.84	±	0.35
USGS66	CN23-08	6	-0.75	±	0.09	41.11	±	0.16

Table S7. Means and standard deviations (1σ) for check standards for all analytical sessions.

Standard	Analytical Session	Number	δ^{13}C (1σ)			δ^{15}N (1σ)		
MET	CN17-08	8	-28.63	±	0.06	-4.95	±	0.05
MET	CN17-16	10	-28.68	±	0.10	-4.95	±	0.14
MET	CN18-10	7	-28.63	±	0.23	-5.01	±	0.09
MET	CN19-07	9	-28.62	±	0.07	-5.09	±	0.14
MET	CN19-09	7	-28.57	±	0.05	-5.25	±	0.11
MET	CN19-10	4	-28.60	±	0.02	-5.03	±	0.11
MET	CN19-11	7	-28.62	±	0.05	-5.15	±	0.12
MET	CN19-12	7	-28.59	±	0.03	-5.00	±	0.15
MET	CN19-20	6	-28.59	±	0.03	-5.08	±	0.24
MET	CN19-21	4	-28.62	±	0.05	-5.21	±	0.21
MET	CN19-22	6	-28.60	±	0.06	-5.06	±	0.17
MET	CN19-24	6	-28.59	±	0.04	-5.13	±	0.15
MET	CN20-05	7	-28.60	±	0.03	-5.01	±	0.05
SRM-1	CN17-08	8	-19.44	±	0.07	1.83	±	0.09
SRM-1	CN17-16	3	-19.40	±	0.01	1.64	±	0.20
SRM-1	CN23-08	4	-19.46	±	0.10	1.89	±	0.05
SRM-2	CN17-08	6	-14.82	±	0.07	15.61	±	0.14
SRM-2	CN17-16	2	-14.77	±	0.05	15.49	±	0.00
SRM-2	CN17-16	2	-14.77	±	0.05	5.21	±	0.15
SRM-3	CN18-10	6	-15.37	±	0.40	21.86	±	0.17
SRM-14	CN21-01	6	-13.62	±	0.05	21.68	±	0.41
SRM-14	CN21-02	6	-13.65	±	0.03	21.57	±	0.13
SRM-14	CN21-03	6	-13.68	±	0.04	21.44	±	0.27
SRM-14	CN22-01	3	-13.61	±	0.03	21.44	±	0.10
SRM-14	CN23-08	4	-13.62	±	0.11	21.29	±	0.10
SRM-15	CN19-02	3	-26.86	±	0.03	6.77	±	0.06
SRM-15	CN19-07	4	-26.90	±	0.04	7.00	±	0.08
SRM-15	CN19-09	6	-26.88	±	0.03	6.92	±	0.08
SRM-15	CN19-10	3	-26.89	±	0.02	7.06	±	0.09
SRM-15	CN19-11	6	-26.86	±	0.04	6.87	±	0.10
SRM-15	CN19-12	5	-26.88	±	0.05	6.98	±	0.19
SRM-15	CN19-20	6	-26.91	±	0.03	6.86	±	0.10
SRM-15	CN19-21	5	-26.89	±	0.04	6.83	±	0.10
SRM-15	CN19-22	5	-26.87	±	0.03	6.93	±	0.13
SRM-15	CN19-24	6	-26.90	±	0.03	6.86	±	0.16
SRM-15	CN20-05	7	-26.89	±	0.03	6.90	±	0.05
SRM-15	CN20-10	4	-26.75	±	0.19	6.83	±	0.06
SRM-15	CN20-13	6	-26.94	±	0.15	6.90	±	0.16

SRM-15	CN21-01	9	-26.90	±	0.04	6.73	±	0.20
SRM-15	CN21-02	9	-26.90	±	0.04	7.06	±	0.46
SRM-15	CN21-03	9	-26.91	±	0.06	6.98	±	0.12
SRM-15	CN22-01	3	-26.92	±	0.02	6.86	±	0.29
SRM-15	CN22-10	4	-26.91	±	0.03	6.95	±	0.03
SRM-15	CN22-13	3	-26.88	±	0.08	6.85	±	0.07
SRM-15	CN22-14	4	-26.92	±	0.04	6.91	±	0.08
SRM-15	CN22-23	3	-26.91	±	0.09	6.86	±	0.02
SRM-15	CN23-05	4	-27.01	±	0.09	6.94	±	0.07
SRM-15	CN23-07	4	-27.05	±	0.05	6.96	±	0.10
SRM-16	CN18-10	5	-14.89	±	0.41	16.90	±	0.08
SRM-16	CN19-07	8	-14.82	±	0.03	17.04	±	0.17
SRM-16	CN19-09	3	-14.78	±	0.07	16.97	±	0.11
SRM-16	CN19-10	4	-14.81	±	0.03	16.80	±	0.32
SRM-16	CN19-11	4	-14.82	±	0.03	17.04	±	0.09
SRM-16	CN19-12	4	-14.80	±	0.03	17.07	±	0.13
SRM-16	CN19-20	5	-14.79	±	0.02	16.90	±	0.09
SRM-16	CN19-21	4	-14.81	±	0.03	17.12	±	0.21
SRM-16	CN19-22	4	-14.77	±	0.09	17.13	±	0.05
SRM-16	CN19-24	5	-14.82	±	0.04	17.10	±	0.18
SRM-16	CN20-05	5	-14.82	±	0.04	17.00	±	0.12
SRM-16	CN20-10	4	-14.68	±	0.17	16.95	±	0.07
SRM-16	CN20-13	5	-14.82	±	0.16	16.99	±	0.21
SRM-16	CN21-01	6	-14.78	±	0.01	17.04	±	0.28
SRM-16	CN21-02	6	-14.78	±	0.02	16.88	±	0.07
SRM-16	CN21-03	6	-14.81	±	0.02	16.98	±	0.02
SRM-16	CN22-01	5	-14.77	±	0.03	16.99	±	0.13
SRM-16	CN22-10	3	-14.81	±	0.02	16.81	±	0.06
SRM-16	CN22-13	4	-14.79	±	0.04	16.73	±	0.07
SRM-16	CN22-14	4	-14.71	±	0.01	16.80	±	0.09
SRM-16	CN22-23	5	-14.67	±	0.08	16.79	±	0.12
SRM-16	CN23-05	4	-14.71	±	0.07	16.77	±	0.14
SRM-16	CN23-07	3	-14.62	±	0.02	16.70	±	0.02
SRM-25	CN21-01	3	-33.20	±	0.06	-13.35	±	0.11
SRM-25	CN21-02	3	-33.25	±	0.04	-12.96	±	0.21
SRM-25	CN21-03	3	-33.20	±	0.03	-12.93	±	0.07
SRM-25	CN22-01	3	-33.25	±	0.02	-12.93	±	0.24
SRM-26	CN22-10	6	-16.17	±	0.07	14.62	±	0.16
SRM-26	CN22-13	7	-16.24	±	0.04	14.67	±	0.12
SRM-26	CN22-14	8	-16.19	±	0.04	14.71	±	0.10
SRM-26	CN22-20	6	-16.20	±	0.06	14.66	±	0.14

SRM-26	CN22-21	4	-16.12	±	0.10	14.67	±	0.11
SRM-26	CN22-23	8	-16.21	±	0.09	14.71	±	0.37
SRM-26	CN23-05	8	-16.13	±	0.10	14.70	±	0.15
SRM-26	CN23-07	6	-16.22	±	0.14	14.66	±	0.12
SRM-26	CN23-08	8	-16.13	±	0.04	14.79	±	0.11
SRM-28	CN22-10	5	-16.30	±	0.11	-1.92	±	0.06
SRM-28	CN22-13	5	-16.22	±	0.06	-1.96	±	0.17
SRM-28	CN22-14	5	-16.23	±	0.05	-1.87	±	0.13
SRM-28	CN22-20	3	-16.32	±	0.23	-1.95	±	0.12
SRM-28	CN22-23	5	-16.26	±	0.13	-1.78	±	0.20
SRM-28	CN23-05	5	-16.32	±	0.09	-1.85	±	0.29
SRM-28	CN23-07	5	-16.21	±	0.04	-1.80	±	0.23
SRM-28	CN23-08	5	-16.33	±	0.15	-1.91	±	0.10
USGS63	CN22-13	6	-1.40	±	0.52	38.05	±	0.12
USGS66	CN21-01	6	-0.73	±	0.16	41.29	±	0.48
USGS66	CN21-02	6	-0.72	±	0.07	40.82	±	0.40
USGS66	CN21-03	6	-0.76	±	0.04	41.04	±	0.22
USGS66	CN22-10	4	-0.73	±	0.08	40.91	±	0.13

Table S8. Means and standard deviations (1σ) for sample replicates for all analytical sessions.

Sample	$\delta^{13}C$ (A)	$\delta^{13}C$ (B)	$\delta^{13}C$ (C)	$\delta^{13}C$ (1σ)	$\delta^{15}N$ (A)	$\delta^{15}N$ (B)	$\delta^{15}N$ (C)	$\delta^{15}N$ (1σ)
HEAL-14	-16.85	-16.89		-16.87 ± 0.03	9.97	10.38		10.18 ± 0.29
HEAL-21	-19.04	-19.06		-19.05 ± 0.01	8.46	8.30		8.38 ± 0.11
HEAL-39	-18.14	-18.21		-18.17 ± 0.05	6.30	6.09		6.20 ± 0.14
HEAL-60	-17.37	-17.41		-17.39 ± 0.03	6.70	6.50		6.60 ± 0.14
HEAL-91	-13.11	-13.20		-13.16 ± 0.06	7.77	7.24		7.51 ± 0.38
HEAL-440	-8.34	-8.33		-8.33 ± 0.01	9.96	10.43		10.19 ± 0.33
HEAL-482	-7.60	-7.59		-7.60 ± 0.01	9.15	9.57		9.36 ± 0.30
HEAL-541	-11.38	-11.34		-11.36 ± 0.03	11.53	11.68		11.61 ± 0.10
HEAL-544	-17.53	-17.51		-17.52 ± 0.01	8.00	8.12		8.06 ± 0.08
HEAL-547	-6.89	-6.75		-6.82 ± 0.10	8.23	8.30		8.26 ± 0.05
HEAL-623	-11.20	-11.21		-11.20 ± 0.01	7.12	6.96		7.04 ± 0.11
HEAL-2306	-18.89	-18.87		-18.88 ± 0.01	12.46	12.43		12.44 ± 0.02
HEAL-2309	-7.00	-7.01		-7.00 ± 0.01	4.76	4.64		4.70 ± 0.09
HEAL-2311	-15.29	-15.18		-15.23 ± 0.08	10.38	10.25		10.31 ± 0.09
HEAL-2313	-18.81	-18.86		-18.83 ± 0.03	9.70	9.70		9.70 ± 0.00
IUBC-2583	-16.67	-16.61		-16.64 ± 0.04	10.34	10.18		10.26 ± 0.12
IUBC-2681	-18.92	-18.95		-18.93 ± 0.02	8.80	8.80		8.80 ± 0.00
TEAL-2862	-16.28	-16.15		-16.21 ± 0.09	11.29	11.42		11.35 ± 0.09
TEAL-2866	-16.73	-16.61		-16.67 ± 0.09	11.23	11.21		11.22 ± 0.01
TEAL-2870	-12.24	-12.25		-12.25 ± 0.01	10.45	10.45		10.45 ± 0.00
TEAL-2876	-16.69	-16.76		-16.73 ± 0.05	10.18	9.78		9.98 ± 0.28
TEAL-3664	-18.04	-18.05		-18.05 ± 0.01	12.73	12.77		12.75 ± 0.03
TEAL-3665	-19.12	-19.17		-19.14 ± 0.03	11.70	11.73		11.71 ± 0.02
TEAL-3666	-19.59	-19.64		-19.62 ± 0.03	10.07	10.11		10.09 ± 0.02
TEAL-3680	-17.50	-17.49		-17.49 ± 0.01	10.79	10.74		10.76 ± 0.04
TEAL-3681	-16.56	-16.59		-16.57 ± 0.02	11.25	11.25		11.25 ± 0.00
TEAL-3685	-18.25	-18.27		-18.26 ± 0.01	11.88	11.88		11.88 ± 0.00
TEAL-3691	-19.67	-19.70		-19.69 ± 0.02	9.86	9.83		9.84 ± 0.02
TEAL-3692	-19.90	-19.97		-19.94 ± 0.05	10.54	10.68		10.61 ± 0.09
TEAL-3724	-18.84	-18.81		-18.82 ± 0.02	8.69	8.90		8.80 ± 0.15
TEAL-3735	-18.78	-18.74	-18.76	-18.76 ± 0.03	10.83	10.90	10.80	10.87 ± 0.05
TEAL-3736	-19.37	-19.30		-19.34 ± 0.05	10.39	10.48		10.43 ± 0.06
TEAL-3737	-19.17	-19.03		-19.10 ± 0.10	10.36	10.26		10.31 ± 0.07
TEAL-3738	-20.12	-19.97		-20.04 ± 0.10	11.00	11.09		11.04 ± 0.06
TEAL-3739	-18.75	-18.65		-18.70 ± 0.07	10.24	10.38		10.31 ± 0.10
TEAL-3740	-19.62	-19.46		-19.54 ± 0.12	9.77	9.65		9.71 ± 0.09
TEAL-3741	-18.65	-18.53		-18.59 ± 0.08	11.14	11.24		11.19 ± 0.07

TEAL-3742	-19.77	-19.67		-19.72 ± 0.07	10.38	10.38		10.38 ± 0.00
TEAL-3743	-20.52	-20.44		-20.48 ± 0.06	10.55	10.49		10.52 ± 0.04
TEAL-3753	-18.13	-18.05		-18.09 ± 0.06	11.11	11.04		11.08 ± 0.05
TEAL-3754	-18.05	-18.02		-18.04 ± 0.02	11.15	11.30		11.23 ± 0.10
TEAL-3755	-19.10	-19.06		-19.08 ± 0.03	10.68	10.74		10.71 ± 0.04
TEAL-3756	-18.91	-18.90		-18.91 ± 0.00	11.14	11.25		11.20 ± 0.07
TEAL-3757	-19.33	-19.29		-19.31 ± 0.03	9.24	9.20		9.22 ± 0.03
TEAL-3763	-19.99	-19.98		-19.98 ± 0.00	10.43	10.53		10.48 ± 0.07
TEAL-3770	-17.73	-17.76		-17.74 ± 0.02	10.18	10.60		10.39 ± 0.30
TEAL-3771	-11.96	-11.98		-11.97 ± 0.01	7.63	7.91		7.77 ± 0.19
TEAL-3772	-16.32	-16.33		-16.32 ± 0.00	10.26	10.31		10.28 ± 0.04
TEAL-3777	-20.00	-19.92		-19.96 ± 0.05	10.76	10.87		10.81 ± 0.08
TEAL-3780	-18.73	-18.73		-18.73 ± 0.00	10.88	10.92		10.90 ± 0.03
TEAL-3781	-19.74	-19.75		-19.75 ± 0.00	11.63	11.66		11.65 ± 0.02
TEAL-3868	-19.77	-19.73		-19.75 ± 0.03	10.55	10.42		10.49 ± 0.09
TEAL-3883	-19.84	-19.81		-19.83 ± 0.02	10.78	10.84		10.81 ± 0.04
TEAL-3884	-18.27	-18.28		-18.28 ± 0.01	11.16	11.08		11.12 ± 0.05
TEAL-3904	-19.84	-19.91		-19.88 ± 0.05	10.10	10.10		10.10 ± 0.00
IUBC-5507	-15.81	-16.01		-15.91 ± 0.14	9.34	9.39		9.37 ± 0.03
IUBC-5509	-16.08	-15.97		-16.02 ± 0.07	9.11	9.11		9.11 ± 0.00
IUBC-5511	-16.96	-17.18		-17.07 ± 0.15	9.05	9.07		9.06 ± 0.01
IUBC-5513	-16.24	-16.56		-16.40 ± 0.22	9.92	9.78		9.85 ± 0.10
IUBC-5515	-16.07	-16.08		-16.07 ± 0.01	8.82	8.87		8.85 ± 0.04
TEAL-6947	-12.08	-12.03		-12.06 ± 0.03	11.03	11.19		11.11 ± 0.11
TEAL-6948	-14.03	-13.94		-13.99 ± 0.06	10.22	10.48		10.35 ± 0.19
TEAL-6975	-13.08	-13.05		-13.06 ± 0.02	9.87	10.10		9.98 ± 0.16
TEAL-7022	-14.72	-14.76		-14.74 ± 0.03	11.56	11.61		11.58 ± 0.04
TEAL-7037	-14.15	-14.20		-14.18 ± 0.04	10.00	10.31		10.15 ± 0.22
TEAL-7043	-13.29	-13.29		-13.29 ± 0.00	8.97	9.17		9.07 ± 0.14
TEAL-9960	-8.20	-8.17		-8.18 ± 0.02	7.50	7.34		7.42 ± 0.12

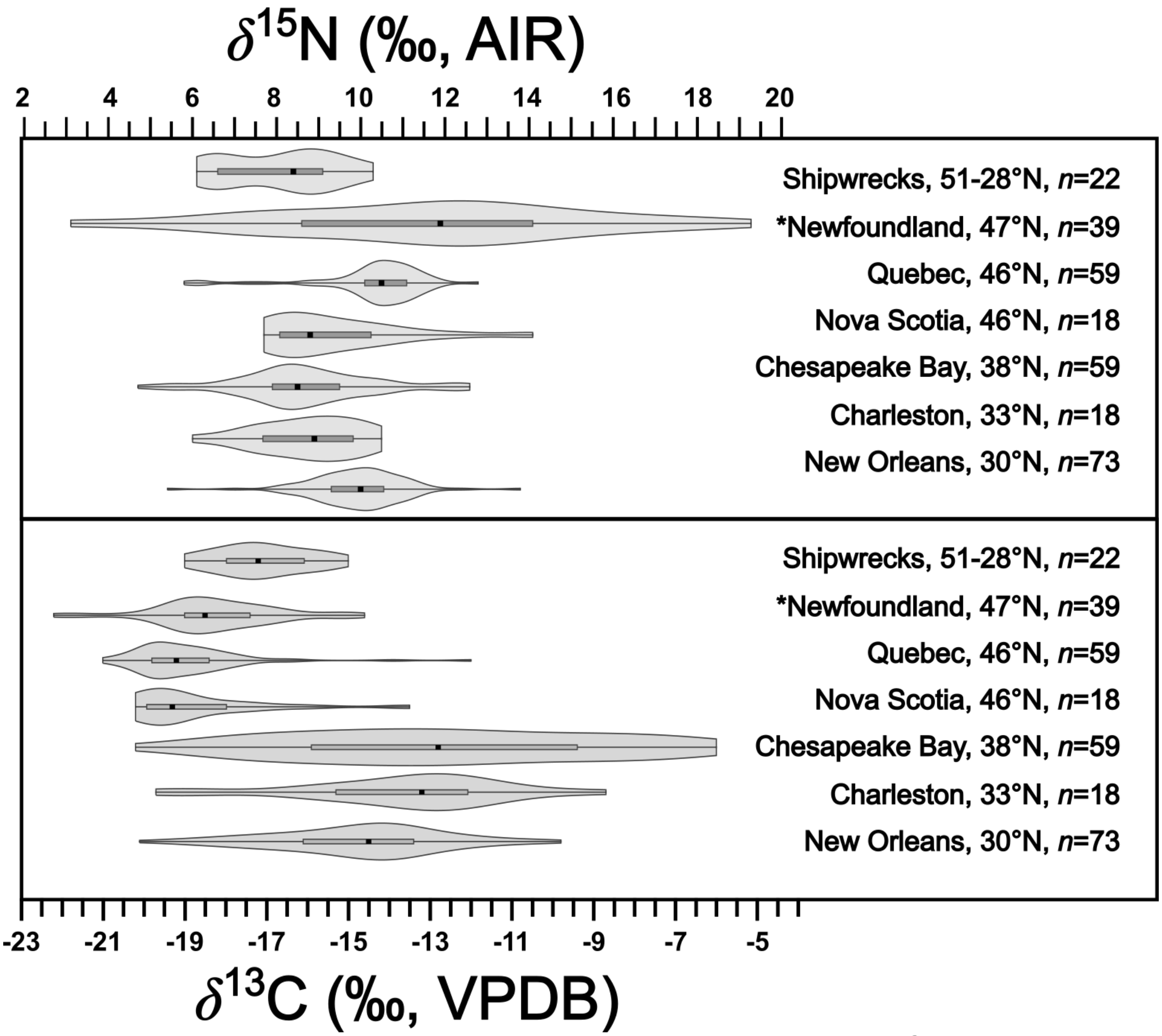

Fig. S1. Violin and box plots showing kernel density for δ^{15}N (upper) and δ^{13}C (lower) grouped by region and ordered by latitude. Asterisks indicate that samples come from a fishing station where higher rat δ^{13}C reflects marine food use. As outlined in the Discussion and Figure 3 (main text), in contrast to δ^{13}C (which, more boradly, reflects changing availability of C_4 derived foods across a latitudinal spectrum), grouping δ^{15}N across taxa is problematic and this comparison if shown here for reference only. Because this plot combines both black and brown rats, which have δ^{15}N values that systematically differ, trends in δ^{15}N are more likely to be driven by the rat species composition observed in a region (see Table S3) than difference in the kinds of foods available in that region.

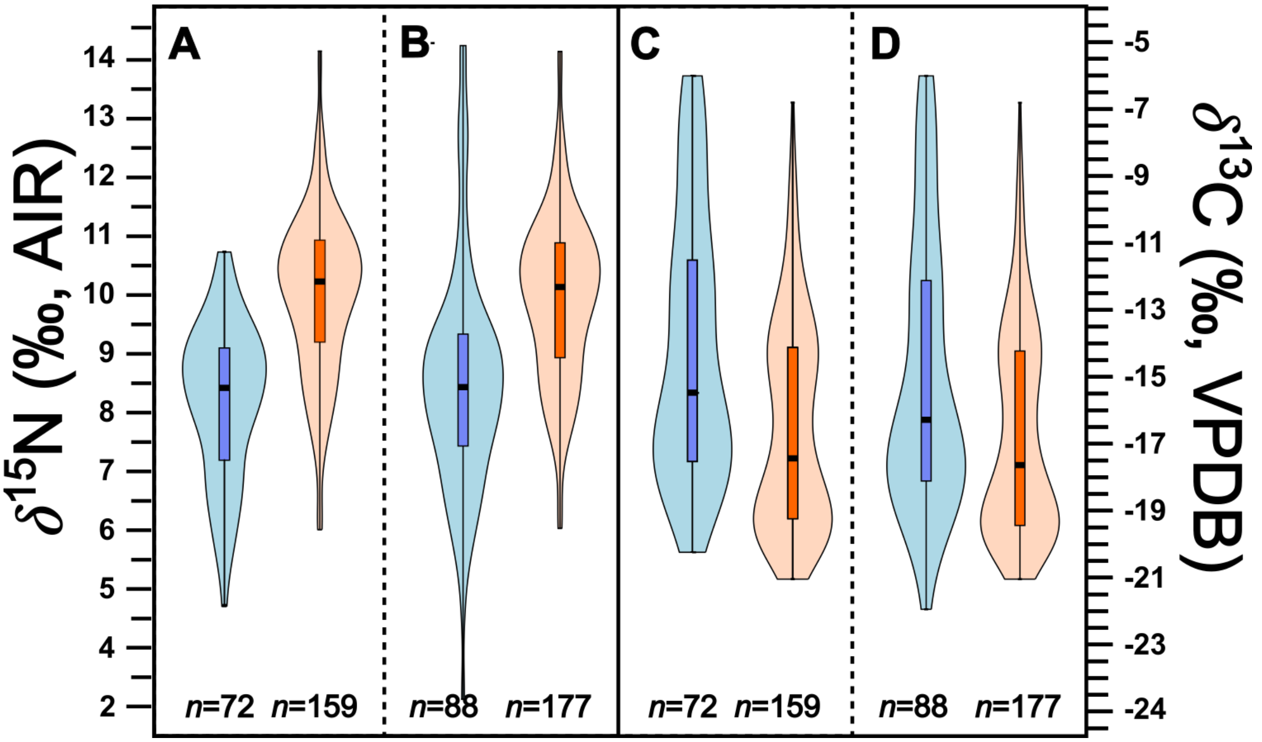

Fig S2. Violin and box plots of isotopic compositions showing kernel density for ZooMS-identified black (blue) and brown (orange) rats for all sites. Panel A shows rat δ^{15}N for all sites excluding fishing stations and panel B shows δ^{15}N for all sites including fishing stations. Panel C shows rat δ^{13}C for all sites excluding fishing stations and panel D shows δ^{13}C for all sites including fishing stations.

REFERENCES AND NOTES

1. P. B. Banks, H. M. Smith, The ecological impacts of commensal species: Black rats, *Rattus rattus*, at the urban–bushland interface. *Wildl. Res.* **42**, 86–97 (2015).

2. A. Hulme-Beaman, K. Dobney, T. Cucchi, J. B. Searle, An ecological and evolutionary framework for commensalism in anthropogenic environments. *Trends Ecol. Evol.* **31**, 633–645 (2016).

3. P. L. Armitage, Commensal rats in the New World, 1492-1992. *Biol. Theory* **40**, 174–178 (1993).

4. P. L. Armitage, Unwelcome companions: Ancient rats reviewed. *Antiquity* **68**, 231–240 (1994).

5. K. P. Aplin, T. Chesser, J. ten Have, Evolutionary biology of the genus *Rattus*: Profile of an archetypal rodent pest, in *Rats, Mice and People: Rodent Biology and Management*, G. R. Singleton, L. A. Hinds, C. J. Krebs, D. M. Spratt, Eds. (Australian Centre for International Agricultural Research, 2003), pp. 487–498.

6. J. Silver, The introduction and spread of house rats in the United States. *J. Mammal.* **8**, 58–60 (1927).

7. N. Bowers, R. Bowers, K. Kaufman, *Kaufman Field Guide to Mammals of North America* (Houghton Mifflin Harcourt, 2007).

8. J. K. Jones, *Handbook of Mammals of the North-Central States* (University of Minnesota Press, 1988).

9. T. L. Best, J. L. Hunt, *Mammals of the Southeastern United States* (University Alabama Press, 2020).

10. D. E. Lantz, *The Brown Rat in the United States* (U.S. Department of Agriculture, Biological Survey, 1909), vol. 33.

11. R. Harlan, *Fauna Americana: Being a Description of the Mammiferous Animals Inhabitating North America* (A. Finley, 1825).

12. M. H. Parsons, P. B. Banks, M. A. Deutsch, R. F. Corrigan, J. Munshi-South, Trends in urban rat ecology: A framework to define the prevailing knowledge gaps and incentives for academia, pest management professionals (PMPs) and public health agencies to participate. *J. Urban Ecol.* **3**, jux005 (2017).

13. A. Y. Feng, C. G. Himsworth, The secret life of the city rat: A review of the ecology of urban Norway and black rats (*Rattus norvegicus* and *Rattus rattus*). *Urban Ecosyst.* **17**, 149–162 (2014).

14. S. P. Foster, *Interspecific Competitive Interactions between* Rattus norvegicus *and* R. rattus (The University of Waikato, 2010).

15. E. J. Guiry, B. C. Gaulton, Inferring human behaviors from isotopic analyses of rat diet: A critical review and historical application. *J. Archaeol. Method Theory* **23**, 399–426 (2016).

16. E. Mori, F. Ferretti, N. Fattorini, Alien war: Ectoparasite load, diet and temporal niche partitioning in a multi-species assembly of small rodents. *Biol. Invasions* **21**, 3305–3318 (2019).

17. W. MacGillivray, *A History of British Quadrupeds: Illustrated by Thirty-four Plates, with Memoir and Portrait of Ulysses Aldrovandi* (W. H Lizars, S. Highley, London; and W. Curry, Jun. and Company, Dublin, 1838), vol. 7.

18. C. M. King, S. Foster, S. Miller, Invasive European rats in Britain and New Zealand: Same species, different outcomes. *J. Zool.* **285**, 172–179 (2011).

19. S. Foster, C. King, B. Patty, S. Miller, Tree-climbing capabilities of Norway and ship rats. *N. Z. J. Zool.* **38**, 285–296 (2011).

20. A. Hulme-Beaman, D. Orton, T. Cucchi, The origins of the domesticate brown rat (*Rattus norvegicus*) and its pathways to domestication. *Anim. Front.* **11**, 78–86 (2021).

21. C. G. Himsworth, K. L. Parsons, C. Jardine, D. M. Patrick, Rats, cities, people, and pathogens: A systematic review and narrative synthesis of literature regarding the ecology of rat-associated zoonoses in urban centers. *Vector Borne Zoonotic Dis.* **13**, 349–359 (2013).

22. C. Diagne, L. Ballesteros-Mejia, R. N. Cuthbert, T. W. Bodey, J. Fantle-Lepczyk, E. Angulo, A. Bang, G. Dobigny, F. Courchamp, Economic costs of invasive rodents worldwide: The tip of the iceberg. *PeerJ* **11**, e14935 (2023).

23. P. L. Armitage, Maps of the continental United States of America showing the chronology and pattern of invasion and range extension of Norway rats Rattus norvegicus in the period c.1745 – 1951. *Zooarchaeol. Res. News* **13**, 16 (1994).

24. R. R. Dunn, J. R. Burger, E. J. Carlen, A. M. Koltz, J. E. Light, R. A. Martin, J. Munshi-South, L. M. Nichols, E. L. Vargo, S. Yitbarek, Y. Zhao, A. Cibrián-Jaramillo, A theory of city biogeography and the origin of urban species. *Front. Conserv. Sci.* **3**, 761449 (2022).

25. A. Desvars-Larrive, M. Baldi, T. Walter, R. Zink, C. Walzer, Brown rats (*Rattus norvegicus*) in urban ecosystems: Are the constraints related to fieldwork a limit to their study? *Urban Ecosyst.* **21**, 951–964 (2018).

26. E. J. Guiry, M. Buckley, Urban rats have less variable, higher protein diets. *Proc. R. Soc. B* **285**, 20181441 (2018).

27. E. E. Puckett, D. Orton, J. Munshi-South, Commensal rats and humans: Integrating rodent phylogeography and zooarchaeology to highlight connections between human societies. *Bioessays* **42**, e1900160 (2020).

28. A. Key, D. Roberts, I. Jarić, Reconstructing the full temporal range of archaeological phenomena from sparse data. *J. Archaeol. Sci.* **135**, 105479 (2021).

29. D. L. Roberts, A. R. Solow, When did the dodo become extinct? *Nature* **426**, 245 (2003).

30. E. J. Guiry, S. Noël, E. Tourigny, V. Grimes, A stable isotope method for identifying transatlantic origin of pig (*Sus scrofa*) remains at French and English fishing stations in Newfoundland. *J. Archaeol. Sci.* **39**, 2012–2022 (2012).

31. C. S. Wan, R. F. Sage, Climate and the distribution of C4 grasses along the Atlantic and Pacific coasts of North America. *Can. J. Bot.* **79**, 474–486 (2001).

32. J. C. Vogel, N. J. van der Merwe, Isotopic evidence for early maize cultivation in New York State. *Am. Antiq.* **42**, 238–242 (1977).

33. G. D. Farquhar, J. R. Ehleringer, K. T. Hubick, Carbon isotope discrimination and photosynthesis. *Annu. Rev. Plant Biol.* **40**, 503–537 (1989).

34. E. J. Guiry, J. C. Hepburn, M. P. Richards, High-resolution serial sampling for nitrogen stable isotope analysis of archaeological mammal teeth. *J. Archaeol. Sci.* **69**, 21–28 (2016).

35. E. Guiry, S. Noël, J. Fowler, Archaeological herbivore δ13C and δ34S provide a marker for saltmarsh use and new insights into the process of 15N-enrichment in coastal plants. *J. Archaeol. Sci.* **125**, 105295 (2021).

36. E. J. Guiry, P. Szpak, M. P. Richards, Isotopic analyses reveal geographical and socioeconomic patterns in historical domestic animal trade between predominantly wheat- and maize-growing agricultural regions in eastern North America. *Am. Antiq.* **82**, 341–352 (2017).

37. E. J. Guiry, B. Harpley, Z. Jones, C. Smith, Integrating stable isotope and zooarchaeological analyses in historical archaeology: A case study from the urban nineteenth-century Commonwealth Block site, Melbourne, Australia. *Int. J. Hist. Archaeol.* **18**, 415–440 (2014).

38. J. A. Swift, Ratting out the past: Investigating anthropogenic landscapes in Polynesia through stable isotope analysis of the Pacific rat (*Rattus exulans*), in *Exploring Human Behavior Through Isotope Analysis: Applications in Archaeological Research*, M. M. Beasley, A. D. Somerville, Eds. (Springer International Publishing, 2023), pp. 225–245.

39. E. J. Guiry, B. M. Jones, S. deFrance, J. E. Bruseth, J. Durst, M. P. Richards, Animal husbandry and colonial adaptive behavior: Isotopic insights from the *La Belle* shipwreck fauna. *Hist. Archaeol.* **52**, 684–699 (2018).

40. E. J. Guiry, J. R. Kennedy, M. T. O'Connell, D. R. Gray, C. Grant, P. Szpak, Early evidence for historical overfishing in the Gulf of Mexico. *Sci. Adv.* **7**, eabh2525 (2021).

41. J. R. Kennedy, E. J. Guiry, Exploring railroad impacts on meat trade: An isotopic investigation of meat sourcing and animal husbandry at Chinese diaspora sites in the American West. *Int. J. Hist. Archaeol.* **27**, 393–423 (2023).

42. E. J. Guiry, T. J. Orchard, S. Needs-Howarth, P. Szpak, Freshwater wetland–Driven variation in sulfur isotope compositions: Implications for human paleodiet and ecological research. *Front. Ecol. Evol.* **10**, 953042 (2022).

43. H. J. Hedrich, Taxonomy and stocks and strains, in *The Laboratory Rat* (Elsevier, 2020), pp. 47–75.

44. H. A. Freye, E. Thenius, in *Grzimeks Tierleben, vol. 11,* B. Grzimek, Ed. (Kindler, 1968), pp. 204–211.

45. P. Migaud, A first approach to links between animals and life on board sailing vessels (1500–1800). *Int. J. Naut. Archaeol.* **40**, 283–292 (2011).
46. C. Dagneau, *État de la recherche concernant la synthèse archéologique du Machault* (Service d'archéologie subaquatique Centre de service de l'Ontario Parcs Canada, 2008).
47. J. D. Godman, P. Maverick, *American Natural History. Volume 3. Part 1—Mastology* (H.C. Carey & I. Lea, 1826), vol. v.2(1826) c.2.
48. J. J. Audubon, J Bachman, *The Viviparous Quadrupeds of North America* (J.J. Audubon, 1846), vol. v.1.
49. M. J. DeNiro, S. Epstein, Influence of diet on the distribution of nitrogen isotopes in animals. *Geochim. Cosmochim. Acta* **45**, 341–351 (1981).
50. M. A. Katzenberg, Stable isotope analysis of archaeological faunal remains from southern Ontario. *J. Archaeol. Sci.* **16**, 319–329 (1989).
51. P. Szpak, Complexities of nitrogen isotope biogeochemistry in plant-soil systems: Implications for the study of ancient agricultural and animal management practices. *Front. Plant Sci.* **5**, 288 (2014).
52. E. Guiry, Complexities of stable carbon and nitrogen isotope biogeochemistry in ancient freshwater ecosystems: Implications for the study of past subsistence and environmental change. *Front. Ecol. Evol.* **7**, 1–24 (2019).
53. Q. Duron, E. Bourguet, M. Thibault, S. Scussel, R. Gouyet, M. Méheut, E. Vidal, Sympatric invasive rats show different diets in a tropical rainforest of an island biodiversity hotspot. *Pac. Sci.* **73**, 199–214 (2019).

54. A. B. Shiels, C. A. Flores, A. Khamsing, P. D. Krushelnycky, S. M. Mosher, D. R. Drake, Dietary niche differentiation among three species of invasive rodents (*Rattus rattus*, *R. exulans*, *Mus musculus*). *Biol. Invasions* **15**, 1037–1048 (2013).

55. G. A. Harper, K. J. Dickinson, P. J. Seddon, Habitat use by three rat species (*Rattus* spp.) on Stewart Island/Rakiura, New Zealand. *N. Z. J. Ecol.* **29**, 251–260 (2005).

56. M. Dammhahn, T. M. Randriamoria, S. M. Goodman, Broad and flexible stable isotope niches in invasive non-native *Rattus* spp. in anthropogenic and natural habitats of central eastern Madagascar. *BMC Ecol.* **17**, 16 (2017).

57. M. W. D. Adams, L. S. Grant, T. G. L. Kovacs, S. Q. T. Liang, N. Norris, H. E. Wesley, M. M. Alessi, P. B. Banks, Commensal black ratsRattus rattusselect wild vegetation over urbanised habitats. *Oikos* **2023**, e09671 (2023).

58. F. Pomeda-Gutiérrez, F. M. Medina, M. Nogales, P. Vargas, Diet of the black rat (*Rattus rattus*) in a Canary laurel forest: Species identification based on morphological markers and DNA sequences. *J. Nat. Hist.* **55**, 629–648 (2021).

59. D. H. Ecke, An invasion of Norway rats in southwest Georgia. *J. Mammal.* **35**, 521–525 (1954).

60. A. C. Peterson, *H. Sharma, A. Kumar, B. M. Ghersi, S. J. Emrich, K. J. Vandegrift, A. Kapoor, M. J. Blum*, Rodent virus diversity and differentiation across Post-Katrina New Orleans. *Sustainability* **13**, 8034 (2021).

61. C. G. Himsworth, C. M. Jardine, K. L. Parsons, A. Y. Feng, D. M. Patrick, The characteristics of wild rat (*Rattus* spp.) populations from an inner-city neighborhood with a focus on factors critical to the understanding of rat-associated zoonoses. *PLOS ONE* **9**, e91654 (2014).

62. J. Gurnell, L. A. Wauters, P. W. W. Lurz, G. Tosi, Alien species and interspecific competition: Effects of introduced eastern grey squirrels on red squirrel population dynamics. *J. Anim. Ecol.* **73**, 26–35 (2004).

63. B. D. Griffen, I. Altman, J. Hurley, H. Mosblack, Reduced fecundity by one invader in the presence of another: A potential mechanism leading to species replacement. *J. Exp. Mar. Biol. Ecol.* **406**, 6–13 (2011).

64. S. Barnett, M. M. Spencer, Feeding, social behaviour and interspecific competition in wild rats. *Behaviour* **3**, 229–242 (1951).

65. S. A. Barnett, in *Proceedings of the Zoological Society of London* (Wiley Online Library, 1958), vol. 130, pp. 107–152.

66. R. L. Williams, G. R. Singleton, C. R. Dickman, The role of interspecific competition in determining macrohabitat use by the black rat and brown rat at Bradley's Head, NSW, in *Rats, Mice and People: Rodent Biology and Management*, G. R. Singleton, L. A. Hinds, C. J. Krebs, D. M. Spratt, Eds. (Australian Centre for International Agricultural Research, 2003), pp. 366–370.

67. E. E. Puckett, J. Munshi-South, Brown rat demography reveals pre-commensal structure in eastern Asia before expansion into Southeast Asia. *Genome Res.* **29**, 762–770 (2019).

68. M.-J. Brisson, A. Humblot, Maisonneuve, *Le regne animal divisé en IX classes, ou, Méthode contenant la division generale des animaux en IX classes : & la division particuliere des deux premieres classes, sçavoir de celle des quadrupedes & de celle des cetacées, en ordres, sections, genres & espéces: Aux quelles on a joint une courte description de chaque espéce, avec les citations des auteurs qui en ont traité, les noms qu'ils les différentes nations, & les noms vulgaires* (Chez Cl. Jean-Baptiste Bauche, A Paris, 1756).

69. A. Seba Locupletissimi rerum naturalium thesauri accurata descriptio, et iconibus artificiosissimis expressio, per universam physices historiam: Opus, cui, in hoc rerum genere, nullum par exstitit. (Apud J. Wetstenium, & Gul. Smith, & Janssonio-Waesbergios, Amstelaedami, 1734), vol. t.2.
70. E. Guiry, T. J. Orchard, S. Needs-Howarth, P. Szpak, Isotopic evidence for garden hunting and resource depression in the Late Woodland of northeastern North America. *Am. Antiq.* **86**, 90–110 (2021).
71. R. L. Lyman, Applied zooarchaeology: The relevance of faunal analysis to wildlife management. *World Archaeol.* **28**, 110–125 (1996).
72. J. Morin, H. Zhang, T. C.A. Royle, C. Speller, M. Alcaide, R. Morin, D. Yang, DNA-based species identification of ancient salmonid remains provides new insight into pre-contact Coast Salish salmon fisheries in Burrard Inlet, British Columbia, Canada. *J. Archaeol. Sci. Rep.* **37**, 102956 (2021).
73. E. J. Guiry, M. James, C. Cheung, T. C. A. Royle, Four millennia of long-term individual foraging site fidelity in a highly migratory marine predator. *Commun. Biol.* **5**, 368 (2022).
74. E. J. Guiry, T. J. Orchard, T. C. Royle, C. Cheung, D. D. Yang, Dietary plasticity and the extinction of the passenger pigeon (*Ectopistes migratorius*). *Quat. Sci. Rev.* **233**, 106225 (2020).
75. P. Szpak, in *Isotope Research in Zooarchaeology: Methods, Applications and Advances* (University Press of Florida Boca Raton, 2022), pp. 248–270.
76. K. A. Byers, M. J. Lee, D. M. Patrick, C. G. Himsworth, Rats about town: A systematic review of rat movement in urban ecosystems. *Front. Ecol. Evol.* **7**, 13 (2019).
77. X. Guo, C. G. Himsworth, M. J. Lee, K. A. Byers, A systematic review of rat ecology in urban sewer systems. *Urban Ecosyst.* **26**, 223–232 (2023).

78. M. J. Lee, K. A. Byers, C. Stephen, D. M. Patrick, R. Corrigan, S. Iwasawa, C. G. Himsworth, Reconsidering the “war on rats”: What we know from over a century of research into municipal rat management. *Front. Ecol. Evol.* **10**, 813600 (2022).

79. M. H. Parsons, C. M. Jardine, M. S. Crowther, C. G. Himsworth, Trends in urban rodent monitoring and mitigation: Improving our understanding of population and disease ecology, surveillance and control. Front. Ecol. Evol. **7**, 522 (2020).

80. C. J. Schell, L. A. Stanton, J. K. Young, L. M. Angeloni, J. E. Lambert, S. W. Breck, M. H. Murray, The evolutionary consequences of human–wildlife conflict in cities. *Evol. Appl.* **14**, 178–197 (2021).

81. B. C. Verrelli, M. Alberti, S. Des Roches, N. C. Harris, A. P. Hendry, M. T.J. Johnson, A. M. Savage, A. Charmantier, K. M. Gotanda, L. Govaert, L. S. Miles, L. R. Rivkin, K. M. Winchell, K. I. Brans, C. Correa, S. E. Diamond, B. Fitzhugh, N. B. Grimm, S. Hughes, J. M. Marzluff, J. Munshi-South, C. Rojas, J. S. Santangelo, C. J. Schell, J. A. Schweitzer, M. Szulkin, M. C. Urban, Y. Zhou, C. Ziter, A global horizon scan for urban evolutionary ecology. *Trends Ecol. Evol.* **37**, 1006–1019 (2022).

82. L. R. Rivkin, J. S. Santangelo, M. Alberti, M. F. J. Aronson, C. W. de Keyzer, S. E. Diamond, M.J. Fortin, L. J. Frazee, A. J. Gorton, A. P. Hendry, Y. Liu, J. B. Losos, J. S. MacIvor, R. A. Martin, M. J. McDonnell, L. S. Miles, J. Munshi-South, R. W. Ness, A. E. M. Newman, M. R. Stothart, P. Theodorou, K. A. Thompson, B. C. Verrelli, A. Whitehead, K. M. Winchell, M. T. J. Johnson, A roadmap for urban evolutionary ecology. *Evol. Appl.* **12**, 384–398 (2019).

83. J. R. Burger, J. G. Okie, I. A. Hatton, V. P. Weinberger, M. Shrestha, K. J. Liedtke, T. Be, A. R. Cruz, X. Feng, C. Hinojo-Hinojo, A. S. M. G. Kibria, K. C. Ernst, B. J. Enquist, Global city densities: Re-examining urban scaling theory. *Front. Conserv. Sci.* **3**, 879934 (2022).

84. K. A. Hobson, R. G. Clark, Assessing avian diets using stable isotopes I: Turnover of 13C in tissues. *Condor* **94**, 181–188 (1992).

85. R. E. M. Hedges, J. G. Clement, C. D. L. Thomas, T. C. O'Connell, Collagen turnover in the adult femoral mid-shaft: Modeled from anthropogenic radiocarbon tracer measurements. *Am. J. Phys. Anthropol.* **133**, 808–816 (2007).

86. S. H. Ambrose, Preparation and characterization of bone and tooth collagen for isotopic analysis. *J. Archaeol. Sci.* **17**, 431–451 (1990).

87. E. J. Guiry, P. Szpak, Improved quality control criteria for stable carbon and nitrogen isotope measurements of ancient bone collagen. *J. Archaeol. Sci.* **132**, 105416 (2021).

88. M. Buckley, M. Gu, S. Shameer, S. Patel, A. T. Chamberlain, High-throughput collagen fingerprinting of intact microfaunal remains; a low-cost method for distinguishing between murine rodent bones. *Rapid Commun. Mass Spectrom.* **30**, 805–812 (2016).

89. C. Clements, M. C. Clements, Package 'sExtinct'. *Conserv. Biol.* **9**, 923–928 (2012).

90. R Core Team, *R: A Language and Environment for Statistical Computing* (R Foundation for Statistical Computing, 2021).

91. H. Qi, T. B. Coplen, H. Geilmann, W. A. Brand, J. Böhlke, Two new organic reference materials for δ13C and δ15N measurements and a new value for the δ13C of NBS 22 oil. *Rapid Commun. Mass Spectrom.* **17**, 2483–2487 (2003).

92. H. Qi, T. B. Coplen, S. J. Mroczkowski, W. A. Brand, L. Brandes, H. Geilmann, A. Schimmelmann, A new organic reference material,l-glutamic acid, USGS41a, for δ13C and

δ15N measurements—A replacement for USGS41. *Rapid Commun. Mass Spectrom.* **30**, 859–866 (2016).

93. A. Schimmelmann, H. Qi, T. B. Coplen, W. A. Brand, J. Fong, W. Meier-Augenstein, H. F. Kemp, B. Toman, A. Ackermann, S. Assonov, A. T. Aerts-Bijma, R. Brejcha, Y. Chikaraishi, T. Darwish, M. Elsner, M. Gehre, H. Geilmann, M. Gröning, J.F. Hélie, S. Herrero-Martín, H. A. J. Meijer, P. E. Sauer, A. L. Sessions, R. A. Werner, Organic reference materials for hydrogen, carbon, and nitrogen stable isotope-ratio measurements: Caffeines, n-alkanes, fatty acid methyl esters, glycines, L-valines, polyethylenes, and oils. *Anal. Chem.* **88**, 4294–4302 (2016).
